# Ethnobotanical secrets of the Baiga tribe in Chhattisgarh Central India

**DOI:** 10.1038/s41598-025-21403-w

**Published:** 2025-10-31

**Authors:** Ramesh Kumar Ahirwar, Diptesh Kumar Bhoi

**Affiliations:** Department of Botany, Guru Ghasidas Vishwavidyalaya, Bilaspur, Chhattisgarh, 495009 India

**Keywords:** Ethnobotany, Traditional knowledge, Baiga tribe, Medicinal plant, Central India, Use value, Drug discovery, Ecology, Plant sciences, Ecology, Environmental social sciences

## Abstract

The ethnobotanical knowledge of indigenous communities represents a vital yet under documented resource for sustainable healthcare and biodiversity conservation. This study explores the traditional medicinal practices of the Baiga tribe and the phytosociological structure of forests surrounding their villages in Bilaspur district, central India. Fieldwork was conducted between January and December 2024 across six villages, using semi-structured interviews with 74 informants (63 males and 11 females) and quadrat-based vegetation sampling. A total of 80 plant species belonging to 75 genera and 42 families were recorded. Fabaceae was the most dominant family with 11 species. Herbs and trees were the most common life forms (36% each), and open land was the primary habitat (46%). Bark was the most frequently used plant part (24%), with paste preparation (43%) and oral administration (77%) being the most preferred methods. Use value (UV) ranged from 0.08 (*Jatropha curcas* L.) to 0.97 (*Azadirachta indica* A.Juss.), while family use values ranged from 0.12 to 0.95 Informant Consensus Factor (ICF) values ranged from 0.92 to 0.97, indicating strong agreement among informants regarding plant usage. Phytosociological analysis revealed *Diospyros melanoxylon* Roxb. as the most ecologically dominant species (IVI = 44.89), followed by *Shorea robusta* C.F.Gaertn. (IVI = 26.33), both of which also hold significant cultural and medicinal value. *Aegle marmelos* (L.) Corrêa and *Azadirachta indica* A.Juss. also showed high IVI values, reflecting their dual ecological and therapeutic roles. Despite the Baiga tribe’s rich medicinal heritage, their knowledge remains underrepresented in academic literature. This study fills a critical gap by documenting their ethnomedicinal practices and highlighting ecologically important species. To support long-term sustainability, we propose conservation strategies such as the establishment of Medicinal Plant Conservation and Development Areas (MPCDAs), community-based training on sustainable harvesting, and inclusion of ethnobotanical knowledge in local education and healthcare systems. These efforts can help preserve both biodiversity and traditional wisdom for future generations.

## Introduction

Throughout history, humans have shared a deep and enduring relationship with plants, relying on them to meet essential daily needs, including food, shelter, energy, medicine, and animal fodder^1,2^. Medicinal plants have been pivotal in traditional healthcare systems, providing treatments for numerous health conditions^3^. This close association with nature has fostered a vast repository of traditional knowledge about medicinal plants. Such knowledge reflects the unique understanding of specific cultures or communities, refined over generations through customary practices^1,4,5^. The use of medicinal plants has remained a cornerstone of traditional healthcare systems since ancient times.

The traditional knowledge of medicinal plants has gained global recognition in recent decades. According to the WHO, 80% of individuals in developing nations rely on traditional medicine practices^4,6,7^. Ancient texts such as the Susruta Samhita, Rigveda, and Charak Samhita provide extensive insights into traditional herbal medicine, reflecting India’s rich cultural and medicinal heritage^8^. The expertise of remote hill communities in folk remedies should be preserved and studied for potential applications in modern drug development. Traditional medicine represents the thoughtful application of indigenous knowledge, offering significant benefits to underprivileged populations and saving lives. Various plants are used in specific combinations based on the ailment being treated, often mixed with other herbs in precise proportions rather than used individually. Most remedies involve crushing plant materials to prepare decoctions, while some are made by boiling specific plant parts in water. These decoctions are typically cooled before oral consumption, with others being directly applied to affected body parts or injuries^9^.

The Baiga tribe, an indigenous community predominantly residing in the central and eastern regions of India, exhibits a rich tapestry of social and cultural values. They are officially recognized as a Scheduled Tribe in multiple states, including Madhya Pradesh, Chhattisgarh, Uttar Pradesh, Jharkhand, and others. The highest population has been recorded in Madhya Pradesh (414,526), followed by Chhattisgarh (89,744), with smaller communities residing in Uttar Pradesh (47,393), West Bengal, Jharkhand, Bihar, Odisha, and Maharashtra^10^. Within Chhattisgarh, the Baiga have been designated as a Particularly Vulnerable Tribal Group (PVTG) due to declining population, low literacy, and limited socio-economic development. Their livelihoods are largely dependent on shifting cultivation (locally known as bewar or dahiya), the collection of forest produce, fishing, and small-game hunting. Ploughing is traditionally avoided as it is believed to injure the Earth, viewed as a sacred maternal entity^11^. These traditional subsistence practices not only reflect deep ecological knowledge but also emphasize the tribe’s sustainable relationship with nature.

These traditions, deeply rooted in their customs, are observed across Madhya Pradesh, Uttar Pradesh, Chhattisgarh, and Jharkhand. Among their myriad cultural practices, the Baigas are particularly recognized for their unique tattooing traditions and distinct rituals associated with birth and death. These traditions form the backbone of their identity and provide insights into their deep connection with nature and community dynamics. One of the most significant aspects of the Baiga culture is their practice of traditional medicine, which remains a vital social value to this day^12–14^. The Baiga men are renowned for their extensive ethnobotanical knowledge, utilizing diverse plant species to prepare herbal remedies for various ailments. Common illnesses such as body pain, headaches, coughs, stomach aches, colds, and fevers, as well as injuries like cuts and bruises, are effectively treated using these indigenous medicinal practices^15,16^. This tradition underscores their intimate understanding of the local flora and highlights the sustainable use of natural resources in their healthcare system. The tribe’s social values are further reflected in their intricate birth and death rituals, which hold profound cultural significance. Postpartum practices designate the mother as impure for a month, culminating in a purification ceremony where the newborn is also named^17–19^. Death rituals reveal a dichotomy; while most individuals are buried, the elderly are cremated as a mark of honour. Corpses are traditionally positioned with their heads pointing south, symbolizing specific spiritual beliefs^20^. In some cases, symbolic items such as coins, cigarettes, or tobacco are placed with the deceased. A coin may even be inserted into the mouth of the dead, later recovered by a close female relative, such as a daughter or sister, who wears it in an amulet^12,21^. These practices not only preserve cultural heritage but also reflect the tribe’s philosophical views on life, death, and the afterlife.

Chhattisgarh, nestled in the heart of India, stands as a beacon of ethnobotanical diversity and a treasure trove of indigenous knowledge. With a rich tribal heritage and sprawling forests covering 44% of its land, the state accounts for an impressive 12% of India’s total forest area. Often celebrated as the “Rice Bowl” of Central India, Chhattisgarh is also home to an extraordinary variety of medicinal plants that have been vital to traditional healthcare systems for generations. Tribal communities such as the Gond, Kawar, Baiga, Abujhmaria, and Kol continue to rely on these natural resources, blending cultural wisdom with sustainable practices to meet their daily needs^22,23^. Among these tribal groups, the Baiga tribe holds a distinct place due to its profound connection with nature and its unparalleled knowledge of medicinal plants. However, despite the significant research on Chhattisgarh’s native flora^24–28^, focused studies on the Baiga tribe remain surprisingly scarce^14,29,30^. This lack of documentation represents a critical gap in understanding the full potential of their ethnobotanical practices and the valuable knowledge they possess about local biodiversity. The Bilaspur district, one of Chhattisgarh’s most culturally vibrant regions, exemplifies the state’s ethnic and ecological richness. According to The 2011 census, tribal communities constitute 14.37% of the district’s population, reflecting a unique blend of traditional wisdom and biodiversity preservation^31^. Yet, systematic research exploring the Baiga tribe’s contributions to this rich heritage has been minimal^14,29,30^.

Although previous ethnobotanical studies have been carried out in neighboring regions of Madhya Pradesh, the present research provides a more comprehensive and in-depth perspective. For example, one study reported only 43 medicinal plant species with limited ethnobotanical data^32^, while another documented 77 species along with basic quantitative observations from the same state^33^. In contrast, the current study was conducted in the Bilaspur district of Chhattisgarh, a region that differs significantly in its cultural and ecological context. Here, we recorded 80 medicinal plant species traditionally used by local communities, supported by detailed quantitative ethnobotanical data. Furthermore, this study uniquely incorporates ecological parameters such as Importance Value Index (IVI), relative density, frequency, and dominance offering fresh insights into the ecological distribution and significance of medicinal plants. These aspects were not addressed in the earlier studies, highlighting the novelty and depth of the present work.

Recognizing this gap, the present study takes a pioneering step to document and analyse the traditional ethnobotanical knowledge of the Baiga tribe in the Bilaspur district. This research delves into their sustainable practices, emphasizing the medicinal significance of native plants and their role in supporting community well-being. By shedding light on these underexplored aspects, the study aims to preserve the Baiga tribe’s unique heritage while unlocking opportunities for its application in modern science and sustainable development. This initiative aspires to position Chhattisgarh as a leading centre of ethnobotanical research in India, celebrating its unparalleled cultural and biological wealth. Through this effort, we not only honour the traditional knowledge of the Baiga tribe but also contribute to the broader understanding and utilization of indigenous practices for a sustainable future.

## Materials and methods

### Study area

Bilaspur, a district rich in history and culture, derives its name from the legendary female fisherman Bilasa, whose legacy dates back over 400 years. Nestled between the coordinates 21º 47’ to 23º 8’ north latitude and 81º 14’ to 83º 15’ east longitude, this district is a geographical and cultural gem in the heart of Chhattisgarh. Bounded by Pendra-Gaurela-Marwahi to the north, Baloda Bazar-Bhatapara to the south, Korba and Janjgir-Champa to the east, and Mungeli and Kabirdham to the west, Bilaspur spans an impressive 3508.48 square kilometers. With a population of 1,625,502, it thrives as a vibrant hub of activity and development. Administratively, Bilaspur is structured into five tehsils and four developmental blocks Belha, Kota, Takhatpur, and Masturi encompassing 708 villages^28^. It proudly holds the distinction of being the second-largest city in the state, following the dynamic Raipur-Bhilai-Durg metropolitan region. Adding to its stature, Bilaspur hosts the Chhattisgarh State High Court in the serene village of Bodri, earning the revered title of “Nyayadhani,” or the Law Capital of Chhattisgarh. As the administrative nerve center of the district, Bilaspur seamlessly blends its historical significance with modern governance, embodying a perfect harmony of heritage and progress^31^.

### Ethnobotanical data collection

**Fig. 1:**
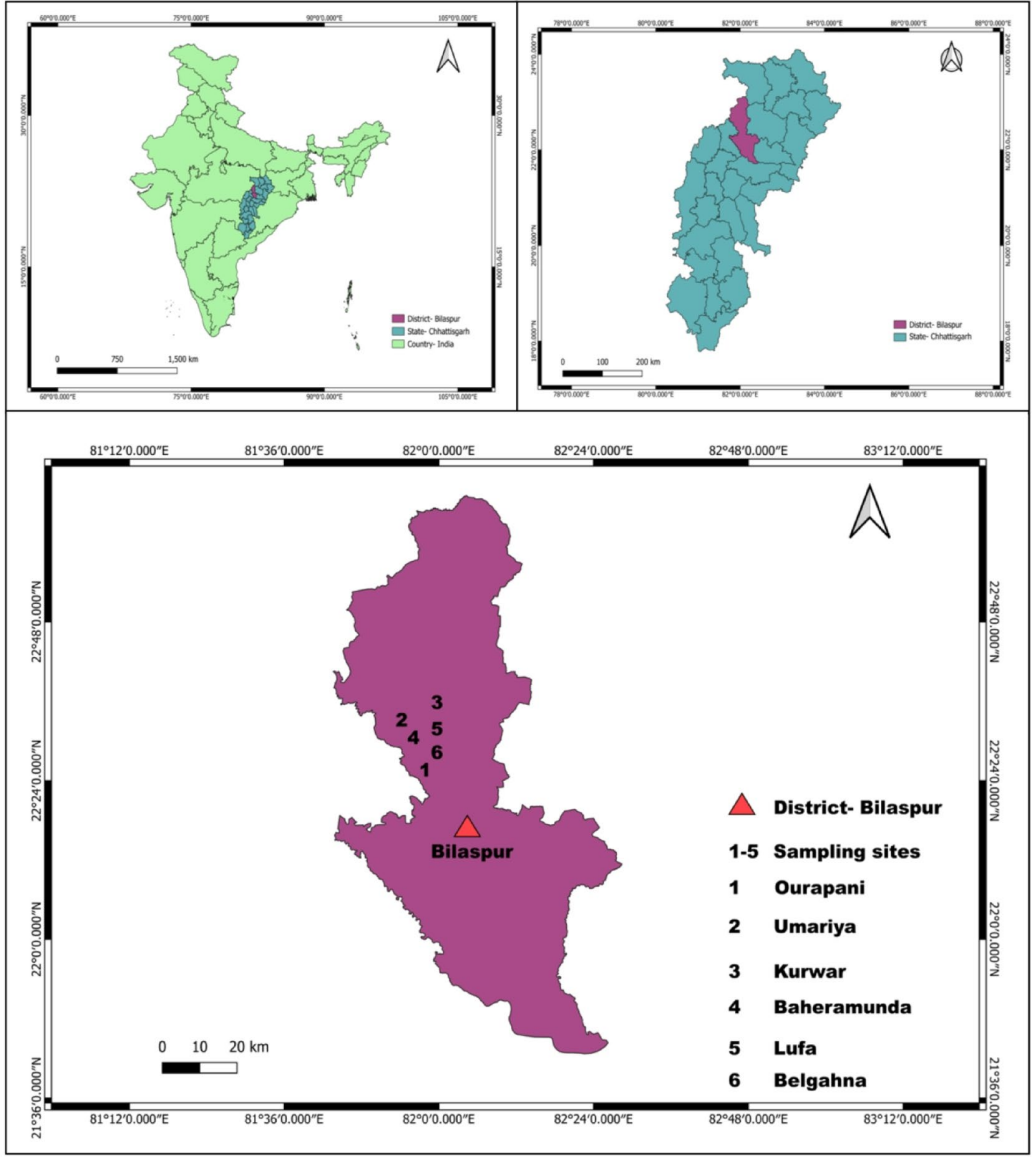
Map of the study site illustrating the geographical location and spatial distribution of the five villages surveyed in the study.

This study was conducted over one year, from January 2024 to December 2024, in six villages of Bilaspur district, namely Ourapani, Umariya, Kurwar, Baheramunda, Lufa, and Belgahna (Fig. 1). The ethnobotanical data were gathered through semi-structured interviews, a method designed to balance structure with flexibility, enabling the collection of in-depth and meaningful information about traditional plant uses^34^. Key informants, such as traditional healers, community elders, and other knowledgeable individuals, were purposefully selected to provide insights into the local uses of plants, including their native names, parts utilized, and practical applications in daily life. The semi-structured format encouraged open-ended dialogue, allowing the informants to share their knowledge freely while ensuring that essential topics were systematically covered. The study adhered to strict ethical guidelines, including obtaining informed consent from participants and respecting their cultural practices and traditions. By employing this approach, the research aimed to capture the essence of indigenous knowledge, offering a detailed understanding of the ethnobotanical practices unique to these communities.

### Ethical considerations

This study was approved by the Institutional Ethical Committee, Guru Ghasidas Vishwavidyalaya, Bilaspur (Reference Number: GGV/IEC/03/014). To safeguard the rights and interests of phytoremedy healers, researchers thoroughly explained the study’s objectives, refrained from offering any incentives, and ensured that participants had the freedom to withdraw at any stage.

### Plant collection and identification

**Fig. 2:**
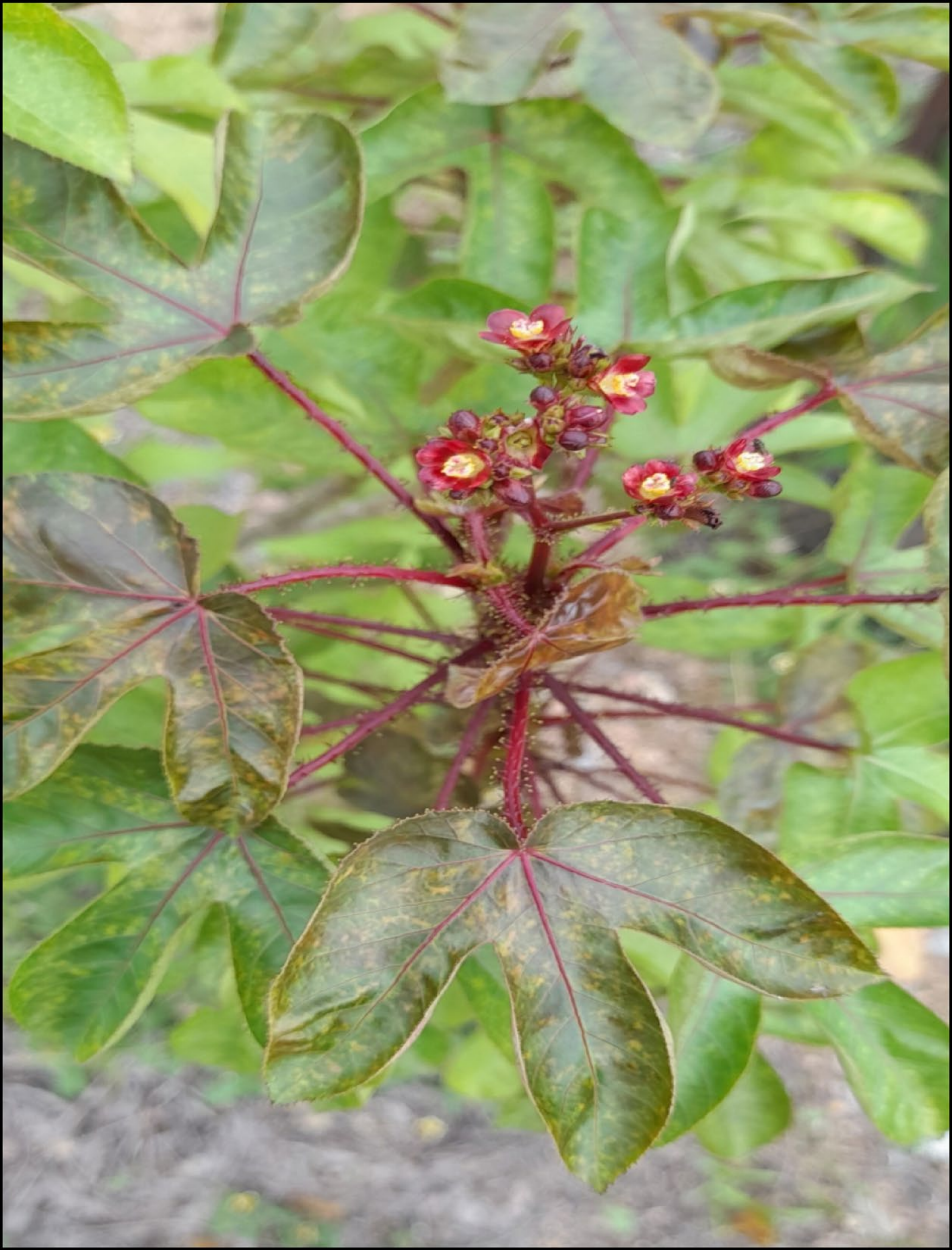
Photograph of *Jatropha gossypiifolia* L.

The collection and identification of plant specimens formed a critical component of this study, ensuring the accuracy and reliability of the documented ethnobotanical information. Plants mentioned during interviews were collected with the guidance of key informants, ensuring alignment between their local names, traditional uses, and the physical specimens (Fig. 2). Field visits were conducted to collect plants from their natural habitats, with specimens primarily gathered during flowering or fruiting stages to aid in precise identification. Each collected specimen was tagged with essential details, including its local name, collection date, habitat description, and the parts used. The specimens were assigned voucher numbers (RKABSP-). The voucher specimens have been deposited in the Herbarium of the Department of Botany, Guru Ghasidas Vishwavidyalaya (A Central University) Bilaspur, Chhattisgarh, India, which is a publicly accessible institutional herbarium. The specimens are preserved following standard herbarium protocols and are available to researchers upon request. The identification of plant specimens was carried out with the assistance of Dr. Ramesh Kumar Ahirwar, at the Department of Botany, Guru Ghasidas Vishwavidyalaya, Bilaspur, Chhattisgarh, India, through a literature survey. The initial identification of plant samples was performed using the British Flora of India, and the Flora of Bilaspur District^35,36^. To ensure further accuracy, the plant names were cross-verified using the Plants of the World Online (POWO) database^37^.

### Use value (UV)

The Use value (UV) method, introduced by^38^, is a robust quantitative approach for determining the relative significance of plant species within a community. By analysing the frequency of plant usage and the number of individuals who report their applications, this method identifies species that hold the greatest importance in traditional practices.

The UV for a plant species is calculated using the following formula:

$$\:UV=\sum\:\frac{Ui}{N}$$

Where:

Ui = represents the total number of use-reports recorded for a particular plant species.

N = is the total number of informants contributing to the study.

A high UV indicates that the plant is widely recognized and frequently utilized, underscoring its cultural or practical significance. In contrast, a UV close to zero suggests that the plant is rarely mentioned, reflecting its lesser role in the community’s ethnobotanical practices. It is important to acknowledge, as noted by^39^, that the UV method does not distinguish between single and multiple uses of a plant. This simplicity, however, ensures the method remains effective and versatile for quantifying plant importance within diverse cultural contexts.

### Family use value (FUV)

The family use value (FUV) is a vital tool for evaluating the importance of plant families, as proposed by^40^, by adapting the concept of use value to the family level, the FUV provides a broader perspective on the cultural and practical significance of plant families within a community.

The FUV is computed using the formula:

$$\:FUV=\sum\:\frac{UVf}{nt}$$

Where:

*UVf = denotes the total Use Value of all species within a specific plant family.*

*nt* = represents the total number of species in that family.

This approach enables the identification of plant families that hold greater importance in the ethnobotanical knowledge of a community. Higher FUV values suggest that the species within a particular family are widely used and recognized, emphasizing their cultural or practical relevance. On the other hand, lower FUV values indicate that the family is less significant in the community’s plant-based knowledge and practices.

### Relative frequency citation (RFC)

The Relative Frequency of Citation (RFC) was calculated using the formula:

$$RFC=\frac{FC}{N}$$

Here, **FC** refers to the number of informants who mentioned a specific plant species as useful, while **N** denotes the total number of informants interviewed during the study. The RFC value ranges from 0 to 1, where 0 indicates that the species was not cited by any informant, and 1 signifies that it was cited by all. This index offers a clear and quantitative measure of the cultural significance and local relevance of each species. The concept was first introduced by Tardío and Pardo-De-Santayana (2008) as a useful tool in ethnobotanical research^41^.

### Informant consensus factor (ICF)

The Informant Consensus Factor (ICF) is a key tool for measuring the level of agreement among informants on the use of plants for purposes, as outlined by^42^ and further refined by^43^.

The ICF is determined using the following formula:

$$\:ICF=\frac{Nur-Nt}{Nur-1}$$

Where:

Nt = represents the total number of plant species reported for a specific ailment or category.

Nur = refers to the total number of usage reports recorded for each disease category.

ICF values range from 0 to 1, providing insights into the consensus level. A value close to zero indicates little agreement among informants, which could be due to varying decisions or limited exchange of knowledge. Conversely, a value near one signifies strong agreement, suggesting that the plant species used for treating certain ailments are widely recognized and consistently applied by community members. This higher consensus reflects the collective and shared understanding of traditional practices^44^.

### Phytosociological study

This study was conducted at different sites within the Ourapani forest of Bilaspur district, Chhattisgarh, India. At designated locations, transects were systematically established, and quadrates were placed along them to assess vegetation composition. A total of ten quadrates, each measuring 10 × 10 m, were laid out at each site to record all individual species present. Data collection and subsequent calculations followed the methodologies outlined by Phillips and Misra^45,46^.

### Analytical characters

$$\:Density=\frac{Total\:number\:individuals\:of\:a\:species}{Total\:number\:of\:quadrates\:studied}$$

$$\:Frequency=\frac{Number\:of\:quadrates\:in\:which\:a\:species\:occurs}{Total\:number\:of\:quadrates\:sampled}X\:100$$

$$\:Abundance=\frac{Total\:number\:individuals\:of\:a\:species\:in\:all\:quadrates}{Total\:number\:of\:the\:quadrates\:in\:which\:the\:species\:occured}\:\:$$

### Synthetic characters

$$\:Relative\:Density=\frac{Number\:of\:individuals\:of\:the\:species}{Total\:number\:of\:individuals\:of\:all\:species}X\:100$$

$$\:Relative\:Frequency=\frac{Number\:of\:occurence\:of\:the\:species}{Number\:of\:occurence\:of\:all\:species}X\:100$$

$$\:Relative\:Dominance=\frac{Total\:basal\:area\:of\:the\:species}{Total\:basal\:area\:of\:all\:species}X\:100$$

### Basal area calculation

We measured the circumference of trees within each quadrate at chest height using a measuring tape. We then determined the radius using the appropriate formula and calculated the basal area of each tree following Misra (1968)^46^, as shown below:

$$\:Basal\:area\:\left(unit{s}^{2}\right)\:\:r=\pi\:\:{\left(\frac{DBH}{2}\right)}^{2}$$

$$\:\pi=3.14$$

$$\:Basal\:area=\:\pi{r}^{2}\:$$

where:

 = 3.14

r = radius of the tree

DBH (Diameter at Breast Height) = Diameter of the tree trunk, usually measured at 1.37 m (4.5 feet) above the ground. If DBH is measured in centimetres, we converted it to meters by dividing by 100.

### Importance value index (IVI)

IVI indicates the ecological significance and dominance of a species in a plant community. Since it considers species density, frequency, and abundance, it serves as a sensitive indicator of anthropogenic activities. We calculated IVI values exclusively for trees and shrubs.

## Results and discussion

### Sociodemographic data of the study area

The sociodemographic profile of the informants provides valuable insights into their composition based on gender, ethnicity, age, education, and occupation. The sample consisted predominantly of males, who accounted for 85% (63 individuals), while females made up only 14% (11 individuals). All the participants belonged to the Baiga ethnic group, indicating a homogeneous ethnic composition that aligns with the focus on this indigenous community. The age distribution reveals a significant representation of younger and middle-aged individuals. A majority (59%) of the informants were between the ages of 24 and 50, followed by 29% in the 51 to 75 age group (Table 1). Only 10% of the respondents were aged 76 to 100, highlighting a balanced inclusion across generational cohorts but with a focus on economically and socially active groups. Education levels among the informants highlight a trend of low formal educational attainment. Over half (55%) of the participants reported never having attended school, while 27% had completed only primary education (classes 1 to 5). A smaller proportion achieved high school education (12%), and only 5% pursued education beyond class 10. This pattern underscores the limited access to formal education within the community, which could reflect broader socio-economic challenges faced by the Baiga people. Occupational data show that the majority (70%) of the participants were farmers, reflecting a heavy reliance on agriculture as the primary source of livelihood. Housewives accounted for 14%, while laborers constituted 10%. A minimal proportion (4%) reported occupations categorized as Other (including teacher and shopkeeper). These findings highlight the community’s agrarian lifestyle and suggest limited diversification in employment opportunities.

**Table 1:** Sociodemographic characteristics of the Baiga tribe in the Bilaspur district, Chhattisgarh.

S.No	Characteristics	Category	Frequency	Percentage
1	Informants	Male	63	85.14
		Female	11	14.86
2	Ethnicity	Baiga	74	100.00
3	Age	24 to 50	44	59.46
		51 to 75	22	29.73
		76 to 100	8	10.81
4	Educational level	Never going to school	41	55.41
		Primary (class 1 to class 5)	20	27.03
		High school (class 8 to class 10)	9	12.16
		Higher education (above class 10)	4	5.41
5	Occupation	Farmer	52	70.27
		Housewives	11	14.86
		Labourer	8	10.81
		Others	3	4.05

### Diversity of medicinal plants

**Fig. 3:**
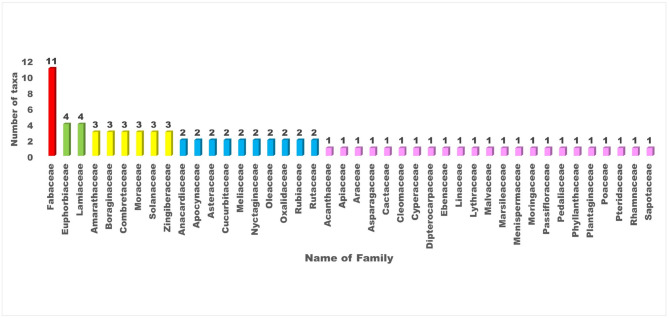
Family distribution of documented medicinal plants.

In this study, a total of 80 plant taxa belonging to 75 genera and 42 families were documented (Table 2). The recorded diversity of plant families highlights the ecological significance and floristic composition of the study area. The Fabaceae family was identified as the most dominant, comprising 11 species. A similar pattern of dominance by Fabaceae was also observed in the neighbouring district of Surguja^47,48^. Globally, Fabaceae ranks third in species diversification^49^. Its dominance in the study area underscores the family’s ecological adaptability and its crucial role in nitrogen fixation, which significantly contributes to soil fertility and ecosystem stability^50^. This predominance aligns with its reported widespread distribution and ecological importance across various ecosystems. Other prominent families include Euphorbiaceae and Lamiaceae, each represented by four species, reflecting their notable contribution to the biodiversity of the region (Fig. 3). These families are recognized for their adaptability to diverse environmental conditions, which may account for their significant representation. Similarly, families such as Amaranthaceae, Boraginaceae, Combretaceae, Moraceae, Solanaceae, and Zingiberaceae, each represented by three species, highlight their ecological importance in maintaining floristic balance. Their presence suggests essential roles in providing ecological services, including pollinator support, fruit production, and habitat stability.

**Table 2:** List of documented medicinal plants recorded in the study area, along with their ethnobotanical applications, utilized plant parts, and modes of preparation.

Family	No. of taxa	Scientific name	Local Name	Plant Habit	Plant Habitat	Plant Class	Plant parts used	Mode of preparation	Ailments treatment	Mode of Administration	Dosages	Precaution	FC	RFC	ΣUi	UV	FUV
Acanthaceae	1	*Ruellia tuberosa* L. [RKABSP-01]	Neeli Neeli	Herb	Forest	Angiosperm	Root	Paste	Cancer (NIC-20), diabetes (NIC-23)	Oral	The Baiga tribe uses a paste prepared from the root of this plant for treating cancer and diabetes. For cancer, a thumb-sized root piece is ground with water, and about one teaspoon of the paste is taken orally once daily for 15–30 days. For diabetes, a smaller amount is used similarly, and half to one teaspoon is consumed orally each morning for 7–15 days.	Although widely practiced, the remedy lacks standardized dosage and scientific safety validation; thus, prolonged use is discouraged, and its administration is not recommended for pregnant women or children.	30	0.41	43	0.58	0.58
Amaranthaceae	1	*Achyranthes aspera* L. [RKABSP-09]	Chirchita	Herb	Open land, road side	Angiosperm	Root	Paste, decoction	Pneumonia (NIC-09), bronchitis (NIC-10), asthma (NIC-05)	External, Oral	The root of this plant is used by the Baiga tribe to treat pneumonia, bronchitis, and asthma through both oral and external applications. A paste is applied externally on the chest in cases of respiratory distress, while a decoction prepared by boiling a small root piece in glass of water until reduced to half is administered orally once daily for 3–5 days.	Though traditionally effective, prolonged use is avoided due to the unknown toxicity profile, and internal administration is not advised for children or pregnant women without medical supervision	12	0.16	24	0.32	0.26
	2	*Gomphrena celosioides* var. celosioides [RKABSP-43]	Gulab chudi	Herb	Open land	Angiosperm	Whole plant	Paste	Constipation (NIC-14)	Oral	A small quantity of whole plant fresh paste is administered orally, usually early in the morning on an empty stomach, for 3 days.	While commonly practiced, dosage is not standardized, and overuse may lead to abdominal discomfort or dehydration; hence, its use should be monitored and avoided in children or during pregnancy.	14	0.19	14	0.19	
	3	*Ouret lanata* (L.) Kuntze [RKABSP-77]	Talmakhana	Herb	Open land	Angiosperm	Whole plant	Paste	Kidney stone (NIC-21)	Oral	Approximately one teaspoon of freshly prepared paste is taken orally once daily, usually in the morning, for 7 to 10 days.	While widely practiced, the remedy is based on traditional knowledge without clinical standardization. Hence, excessive or prolonged use is avoided due to the possibility of gastrointestinal irritation or unknown nephrotoxic effects, and it is not recommended for pregnant women, children, or individuals with pre-existing renal conditions.	21	0.28	21	0.28	
Anacardiaceae	1	*Mangifera indica* L. [RKABSP-90]	Aama	Tree	Open land, forest	Angiosperm	Bark	Paste	Tympanitis (NIC-27), liver problem (NIC-13), heat strokes (NIC-10), bladder catarrh (NIC-7)	External, Oral	A paste is applied externally around the ear to treat tympanitis. For liver problems, heat stroke, and bladder catarrh, the paste is taken orally once daily for 5–7 days.	Though widely used in traditional medicine, internal administration is approached cautiously due to the absence of clinical safety data and potential hepatic or renal risks; hence, it is not advised for children, pregnant women, or individuals with chronic organ conditions.	32	0.43	57	0.77	0.52
	2	*Semecarpus anacardium* L.f. [RKABSP-85]	Bhelwa	Tree	Forest, open land	Angiosperm	Root	Decoction	Cancer (NIC-21)	Oral	A decoction is prepared by boiling the root in water until it reduces to about one cup. This is consumed orally once a day, usually in the morning, for a period of two to four weeks.	The remedy is administered strictly under the guidance of experienced tribal practitioners. Despite its cultural significance, there is no scientific validation of its anticancer efficacy, and internal use is generally discouraged in pregnant women, children, and individuals with serious health conditions.	21	0.28	21	0.28	
Apiaceae	1	*Foeniculum vulgare* Mill. [RKABSP-02]	Saunf	Herb	Home garden	Angiosperm	Whole plant	Paste	Diarrhoea (NIC-25)	Oral	A fresh paste is prepared by crushing the entire plant, and about one to two teaspoons of this paste are taken orally once or twice a day, typically for two to three days or until symptoms subside.	The treatment is generally administered under the guidance of traditional healers. While this remedy holds cultural value, its pharmacological efficacy remains unverified, and caution is advised, particularly in young children, pregnant women, and individuals with chronic gastrointestinal issues.	25	0.34	25	0.34	0.34
Apocynaceae	1	*Calotropis gigantea* (L.) W.T.Aiton [RKABSP-39]	Aankphuti	Shrub	Open land	Angiosperm	Latex	Raw	Swollen (NIC-29)	External	A small amount of fresh latex is directly applied over the swollen area once or twice daily, usually until relief is observed. This practice is performed with care, as the latex may cause irritation or burning in sensitive individuals.	Its use is strictly external, and application on broken skin or near the eyes is avoided. Despite its regular use in traditional healing, scientific evidence supporting its anti-inflammatory activity is limited.	29	0.39	29	0.39	0.27
	2	*Nerium oleander* L. [RKABSP-96]	Kaner	Shrub	Open land	Angiosperm	Root	Decoction	Cancer (NIC-12)	Oral	A decoction is prepared by boiling the root in water until the quantity reduces to about one cup. This is consumed orally once a day, usually in the early morning, for a period as advised by the traditional healer, often extending over several weeks.	The remedy is considered potent and is used only under the careful supervision of experienced practitioners. Owing to the absence of scientific validation and potential toxicity risks, internal use is not recommended for pregnant women, children, or individuals with chronic health conditions.	12	0.16	12	0.16	
Araceae	1	*Colocasia esculenta* (L.) Schott [RKABSP-22]	Kochai, Taro	Herb	Home garden	Angiosperm	Leaves	Paste	Asthma (NIC-6), insect bite (NIC-3)	Oral, External	A fresh paste is prepared by crushing the leaves. For asthma, about one teaspoon of the paste is taken orally once daily, usually in the morning, for a few days under the guidance of a traditional healer. In case of insect bites, the same paste is applied externally over the affected area two to three times a day until swelling and irritation subside.	Though commonly practiced, there is limited scientific evidence supporting its efficacy, and internal use is avoided in children, pregnant women, and those with respiratory complications unless supervised.	7	0.09	9	0.12	0.12
Asparagaceae	1	*Asparagus racemosus* Willd. [RKABSP-08]	Satavar	Herb	Forest, open land	Angiosperm	Tuber	Decoction	Cancer (NIC-13) tuberculosis (NIC-15), females problem (NIC-23)	Oral	The tuber of this plant is used by the Baiga tribe in the treatment of cancer, tuberculosis, and various female reproductive disorders. A decoction is prepared by boiling sliced tubers in water until the volume reduces to about one cup. This preparation is taken orally once daily, typically in the early morning, for a period one to several weeks depending on the condition.	The remedy is administered under close supervision due to the strength of its effects. Despite its cultural importance, there is no scientific evidence validating its efficacy or safety, and it is not recommended for pregnant women, children, or those with liver or kidney ailments.	38	0.51	51	0.69	0.69
Asteraceae	1	*Tridax procumbens* L. [RKABSP-61]	Ghamra	Herb	Open land	Angiosperm	Whole plant	Paste	Diarrhoea (NIC-21), liver problem (NIC-18)	Oral	The whole plant is traditionally used to manage diarrhoea and liver-related ailments. A fresh paste is prepared by crushing the entire plant, and about one to two teaspoons are taken orally once or twice a day, usually before meals, for two to five days depending on the severity of symptoms.	This remedy is generally administered under the guidance of experienced traditional healers. While deeply rooted in indigenous knowledge, its internal use lacks scientific validation and is not recommended for pregnant women, children, or individuals with existing hepatic conditions without proper supervision.	29	0.39	39	0.53	0.62
	2	*Xanthium strumarium* L. [RKABSP-99]	Chhota Gokhru	Herb	Open land, road side	Angiosperm	Root	Paste	Epilepsy (NIC-13), leucoderma (NIC-30), gastric ulcer (10)	Oral	The root of this plant is used by the Baiga tribe in the treatment of epilepsy, leucoderma, and gastric ulcer. A fresh paste is prepared from the root and taken orally about one teaspoon once or twice a day—usually in the morning, under the supervision of traditional healers. The duration of administration varies depending on the condition, often extending from several days to a few weeks.	Although widely used in indigenous healthcare, there is no clinical validation for its efficacy or safety. Internal use is avoided in children, pregnant women, and individuals with chronic neurological or gastrointestinal conditions unless guided by experienced practitioners.	40	0.54	53	0.72	
Boraginaceae	1	*Cordia dichotoma* G.Forst. [RKABSP-35]	Bakain	Tree	Open land, home garden	Angiosperm	Bark, fruit	Decoction, raw	Asthma (NIC-33), urinary tract infection (NIC-14)	Oral	For asthma, a decoction is prepared by boiling the bark in water until it reduces to about one cup. This is taken orally once daily, usually in the morning. In the case of urinary tract infections, the fresh fruit is consumed raw typically one fruit once or twice a day under the guidance of traditional healers.	The remedy is valued in local practices; however, no pharmacological validation exists, and internal use is discouraged in pregnant women, children, and individuals with kidney or respiratory conditions without expert supervision.	35	0.47	47	0.64	0.61
	2	*Cordia macleodii* (Griff.) Hook.f. & Thomson [RKABSP-05]	Dahiman	Tree	Forest	Angiosperm	Bark	Paste	Jaundice (NIC-35)	Oral	A fresh paste is prepared by grinding the bark, and about one to two teaspoons are taken orally once daily, preferably on an empty stomach, for five to seven days or as prescribed by the traditional healer.	This remedy is widely practiced in the region; however, its safety and efficacy have not been scientifically validated. Internal use is avoided in pregnant women, children, and individuals with severe liver conditions unless administered under expert supervision.	35	0.47	35	0.47	
	3	*Heliotropium indicum* L. [RKABSP-87]	Hathajori	Herb	Open land	Angiosperm	Whole plant	Paste	Febrifuge (NIC-22), bone fracture (NIC-32)	Oral, External	A fresh paste is prepared by crushing the entire plant. For fever, about one to two teaspoons of the paste are taken orally once daily, typically in the morning, for two to three days or until the fever subsides. In the case of bone fractures, the paste is applied externally over the affected area, often in combination with traditional splinting methods, and is renewed daily until healing progresses.	While these practices are deeply rooted in tribal medicine, scientific validation is lacking. Caution is advised, particularly with oral use in children, pregnant women, and those with chronic conditions.	33	0.45	54	0.73	
Cactaceae	1	*Opuntia dillenii* (Ker Gawl.) Haw. [RKABSP-93]	Nag pheni	Shrub	Home garden	Angiosperm	Root	Decoction	Asthma (NIC-9), neuron problem (NIC-23)	Oral	The root of the plant is traditionally used by the Baiga community for the treatment of asthma and neurological disorders. A decoction is prepared by boiling the root in water until the volume is reduced to approximately one cup. This is administered orally once daily, typically in the morning, for a duration ranging from 7 to 15 days, depending on the severity of the condition and the advice of traditional healers.	While commonly practiced, the remedy lacks pharmacological validation, and its use is not recommended in children, pregnant women, or individuals with chronic respiratory or neurological conditions without expert guidance.	25	0.34	32	0.43	0.43
Cleomaceae	1	*Cleome viscosa* L. [RKABSP-70]	Bagra	Herb	Open land	Angiosperm	Whole plant	Infusion, paste	Hypertension (NIC-11), wound healing (NIC-12)	Oral, External	For hypertension, an infusion is prepared by soaking the whole plant in warm water, which is taken orally once daily, typically in the morning, for a period of 10 to 15 days. For wound healing, a fresh paste is prepared from the same plant and applied externally over the affected area twice daily until recovery, usually within 5 to 7 days.	Although these practices are widely adopted in local healthcare systems, their efficacy and safety remain unvalidated by scientific studies. Oral use is not advised in children, pregnant women, or individuals with cardiovascular or metabolic disorders without proper supervision.	17	0.23	23	0.31	0.31
Combretaceae	1	*Combretum indicum* (L.) DeFilipps [RKABSP-12]	Madhumalti	Climber	Home garden	Angiosperm	Seed, fruit	Raw	Worms (NIC-06), indigestion (NIC-05)	Oral	For deworming, a small handful of seeds or one fresh fruit is taken raw once daily in the early morning, typically for three to five days. For indigestion, the raw fruit is consumed once or twice daily, generally for two to three days, depending on the severity.	These remedies are commonly practiced under the guidance of local healers. However, in the absence of scientific validation, oral use is not recommended in children, pregnant women, or individuals with chronic digestive conditions without appropriate supervision.	10	0.14	11	0.15	0.54
	2	*Terminalia bellirica* (Gaertn.) Roxb. [RKABSP-06]	Bahera	Tree	Forest	Angiosperm	Bark	Decoction	Asthma (NIC-31), hepatitis (NIC-25)	Oral	A decoction is prepared by boiling the bark in water until the volume is reduced, and is taken orally once daily, preferably in the early morning. For asthma, it is typically administered for 7 to 10 days, while in the case of hepatitis, the duration may extend up to 15 days.	Despite its cultural significance, this practice lacks scientific validation. Oral use is not recommended for children, pregnant women, or individuals with chronic respiratory or hepatic conditions without appropriate supervision.	42	0.57	56	0.76	
	3	*Terminalia chebula* Retz. [RKABSP-30]	Harida	Tree	Forest	Angiosperm	Bark, fruit	Paste	Diabetes (NIC-34), jaundice (NIC-19)	Oral	A paste is prepared by crushing equal portions of bark and fruit, and approximately one to two teaspoons of this mixture are taken orally once daily, usually in the morning. For diabetes, the administration may continue for 15 to 21 days, while for jaundice it is typically given for 7 to 10 days, depending on the condition.	Although these practices are well-established in traditional medicine, no pharmacological validation exists. Oral use is discouraged in children, pregnant women, and individuals with severe hepatic or metabolic disorders unless guided by a qualified practitioner.	44	0.59	53	0.72	
Cucurbitaceae	1	*Coccinia grandis* (L.) Voigt [RKABSP-10]	Kundri	Climber	Home garden	Angiosperm	Leaves	Paste	Epilepsy (NIC-6), anorexia (NIC-37)	Oral	A fresh paste is prepared by grinding the leaves, and approximately one to two teaspoons are taken orally once daily, preferably in the morning on an empty stomach. For epilepsy, the paste is administered for 15 to 21 days, whereas for anorexia, a shorter duration of 5 to 7 days is commonly followed.	These uses are guided by local healers and are embedded in traditional medical practices. However, no scientific evidence currently supports their safety or efficacy. Oral use is not recommended in vulnerable populations, including children and pregnant women, without proper supervision.	37	0.50	43	0.58	0.68
	2	*Momordica dioica* Roxb. ex Willd. [RKABSP-03]	Kaksa	Climber	Home garden, forest	Angiosperm	Leaves	Infusion	Diabetes (NIC-59)	Oral	An infusion is prepared by steeping the leaves in warm water for several minutes. The liquid is consumed orally once daily, preferably in the morning before meals. The treatment duration typically ranges from 15 to 21 days, depending on the patient’s condition.	Although widely used in local health traditions, this remedy lacks pharmacological validation. Its use is not recommended for children, pregnant women, or individuals with chronic metabolic disorders without appropriate supervision.	59	0.80	59	0.80	
Cyperaceae	1	*Cyperus rotundus* L. [RKABSP-88]	Motha	Herb	Open land	Angiosperm	Root	Decoction	Pyrosis (NIC-27), diarrhoea (NIC-40)	Oral	A decoction is prepared by boiling the root in water until the volume reduces to a cup. This preparation is taken orally once daily, preferably in the morning on an empty stomach. For pyrosis, the decoction is usually administered for 3 to 5 days, while for diarrhoea it is given for a shorter duration of 2 to 3 days. These practices are guided by local healers and are rooted in community-based healthcare traditions.	However, in the absence of clinical validation, oral use is not advised for children, pregnant women, or individuals with chronic gastrointestinal conditions without appropriate supervision.	46	0.62	67	0.91	0.91
Dipterocarpaceae	1	*Shorea robusta* C.F.Gaertn. [RKABSP-04]	Sal	Tree	Forest	Angiosperm	Bark	Decoction	Inflammation (NIC-27), ear pain (NIC-13), itching (NIC-30)	External	A decoction is prepared by boiling the bark in water until the liquid is reduced to approximately one cup. Once cooled to a lukewarm temperature, the decoction is applied externally over the affected area using a clean cloth or cotton. For inflammation and itching, it is applied twice daily for 3 to 5 days, while for ear pain, warm drops of the decoction are placed near the outer ear region once or twice daily for 2 to 3 days.	These remedies are administered under the guidance of traditional healers. However, no pharmacological validation exists, and external use should be approached with caution, especially in individuals with skin sensitivities or open wounds.	44	0.59	70	0.95	0.95
Ebenaceae	1	*Diospyros melanoxylon* Roxb. [RKABSP-07]	Tendu	Tree	Open land	Angiosperm	Leaves, bark	Paste, decoction	Scabies (NIC-12), dyspepsia (NIC-19), anemia (NIC-08)	External, Oral	For scabies, a fresh paste is prepared from the leaves and bark and applied externally over the affected skin twice daily for 5 to 7 days, typically until symptoms subside. For dyspepsia and anemia, a decoction is prepared by boiling equal parts of leaves and bark in water and taken orally once daily in the early morning. The duration of oral administration ranges from 7 to 10 days for dyspepsia and up to 15 days for anemia, depending on the severity.	Though widely used, these practices remain unvalidated by modern pharmacological studies, and internal use is not advised for pregnant women, children, or individuals with chronic gastrointestinal or hematological disorders without supervision.	25	0.34	39	0.53	0.53
Euphorbiaceae	1	*Acalypha indica* L. [RKABSP-14]	Muktajhuri	Herb	Open land, road side	Angiosperm	Leaves	Paste	Skin infection (NIC-15)	External	A fresh paste is prepared by grinding the leaves and is applied directly over the affected area twice daily, preferably in the morning and evening. The treatment is continued for 3 to 5 days or until visible improvement occurs, under the guidance of traditional healers.	Although widely practiced, this remedy lacks pharmacological validation. Caution is advised during application over open wounds or sensitive skin.	15	0.20	15	0.20	0.18
	2	*Euphorbia hirta* L. [RKABSP-74]	Dudhi, Doodhiya	Herb	Open land	Angiosperm	Whole plant	Infusion	Coryza (NIC-3), gonorrhea (NIC-7), dengue fever (NIC-11)	Oral	An infusion is prepared by soaking the chopped plant in warm water and allowing it to steep. The liquid is consumed orally once or twice daily, usually on an empty stomach. For coryza, the infusion is taken for 3 to 5 days; for gonorrhea, the treatment continues for 7 to 10 days; and in the case of dengue fever, it is typically administered for 5 to 7 days under the supervision of traditional healers.	Though rooted in local ethnomedicinal knowledge, this remedy lacks scientific validation, and oral intake should be avoided in children, pregnant women, and patients with severe underlying conditions without proper guidance.	16	0.22	21	0.28	
	3	*Jatropha curcas* L. [RKABSP-25]	Ratanjot	Shrub	Open land	Angiosperm	Bark	Paste	Cancer (NIC-6)	Oral	A paste is prepared by grinding the bark with water or cow milk, and is taken orally once daily, preferably in the early morning for the treatment of cancer. The administration is continued for 15 to 21 days depending on the condition and advice of local healers.	While this practice is culturally significant, it remains unsupported by scientific or clinical validation. Due to potential toxicity and unknown side effects, internal use is discouraged in vulnerable individuals, including children, pregnant women, and patients with liver or kidney impairment, without expert supervision.	6	0.08	6	0.08	
	4	*Jatropha gossypiifolia* L. [RKABSP-45]	Ratanjoti	Shrub	Open land	Angiosperm	Bark	Paste	Influenza (NIC-10)	Oral	A paste is prepared by grinding the bark with a small amount of water, and approximately one to two teaspoons are taken orally once daily, preferably on an empty stomach in the morning. The treatment usually continues for 5 to 7 days, or until symptoms subside, under the guidance of local healers.	Though widely practiced in traditional medicine, this remedy has not been scientifically validated. Caution is advised for children, pregnant women, and individuals with compromised immunity or respiratory conditions.	10	0.14	10	0.14	
Fabaceae	1	*Abrus precatorius* L. [RKABSP-64]	Gumchi	Climber	Forest	Angiosperm	Leaves, seed	Infusion, paste	Fever (NIC-20),sores (NIC-9)	Oral, External	For fever, an infusion is prepared by soaking crushed leaves and seeds in warm water, which is then taken orally once daily, preferably in the morning before meals. The treatment usually continues for 3 to 5 days. For sores, a paste is made by grinding the seeds and leaves into a fine consistency and applied externally over the affected area twice daily for 4 to 7 days.	These remedies are practiced under the guidance of local healers. Despite their traditional value, these applications lack pharmacological validation, and caution is advised, particularly in children, pregnant women, and individuals with severe systemic illness.	22	0.30	29	0.39	0.46
	2	*Bauhinia variegata* L. [RKABSP-47]	Koiralo	Tree	Open land, home garden	Angiosperm	Bark	Decoction	Diabetes (NIC-10)	Oral	The bark of this plant is traditionally used by the Baiga community for managing diabetes (NIC-10). A decoction is prepared by boiling small pieces of the bark in water until the volume reduces to approximately one cup. This is consumed orally once daily, preferably in the morning before food. The treatment generally continues for 15 to 21 days under the supervision of traditional healers.	While this remedy is widely practiced in ethnomedicine, it remains unverified by modern pharmacological studies. Therefore, its prolonged use should be approached cautiously, especially in pregnant women, children, and individuals with liver or kidney ailments.	10	0.14	10	0.14	
	3	*Butea monosperma* (Lam.) Kuntze [RKABSP-66]	Palash	Tree	Open land, forest	Angiosperm	Bark	Paste	Keratitis (NIC-13), itch (NIC-20)	External	The paste is obtained by grinding fresh bark with water, producing a thick, smooth preparation. For keratitis, the paste is gently applied around the closed eyelids once daily with clean hands or cloth, avoiding direct contact with the eyes. In cases of itching, it is applied over the affected skin twice daily, typically for 3 to 5 days.	This remedy is guided by traditional knowledge passed down through generations. Despite its popularity, no clinical studies have validated its efficacy or safety; hence, application on sensitive or broken skin should be done with care.	23	0.31	33	0.45	
	4	*Clitoria ternatea* L. [RKABSP-92]	Aparajita	Climber	Home garden	Angiosperm	Flower	Raw	Diabetes (NIC-51)	Oral	Typically, a few fresh flowers are chewed on an empty stomach early in the morning, with the belief that regular intake helps control blood sugar levels. This practice may continue for 10 to 15 days.	Though widely accepted within the community, this usage has not been validated by scientific research. Caution is recommended for individuals with known metabolic or allergic conditions.	51	0.69	51	0.69	
	5	*Dalbergia latifolia* Roxb. [RKABSP-51]	Sheesham	Tree	Forest	Angiosperm	Root	Decoction	Leprosy (NIC-60)	Oral	Within the Baiga ethnomedicinal tradition, the roots of this plant are decocted and administered orally in the treatment of leprosy. The preparation involves boiling crushed roots in water until it reduces to a drinkable amount. The decoction is taken once daily, generally in the morning before food, and the course may extend from two to three weeks depending on the condition.	This traditional remedy reflects deep cultural belief but lacks scientific evidence. Prolonged consumption without supervision is discouraged, especially in individuals with chronic skin disorders, liver issues, or during pregnancy.	60	0.81	60	0.81	
	6	*Delonix regia* (Bojer ex Hook.) Raf. [RKABSP-26]	Gulmohar	Tree	Open land	Angiosperm	Bark	Paste	Wound healing (NIC-17)	External	The Baiga community employs the bark of this plant in wound care practices. A fine paste is prepared by pounding the bark with clean water, which is then gently applied over wounds once or twice a day. The application typically continues for 4 to 6 days or until the wound shows signs of healing.	This method is believed to reduce inflammation and promote tissue repair. Despite its cultural relevance, the remedy has not undergone pharmacological validation, and its use over deep or infected wounds should be approached with caution.	17	0.23	17	0.23	
	7	*Phanera vahlii* (Wight & Arn.) Benth. [RKABSP-32]	Malvan	Shrub	Forest	Angiosperm	Leaves, bark	Paste	Cuts (NIC-42)	External	For treating fresh cuts, the Baiga healers prepare a paste using both the leaves and bark of the plant. The ingredients are crushed together with clean water or occasionally with cow ghee to form a smooth paste. This is directly applied over the cut surface to stop bleeding and promote faster healing. The application is repeated twice daily for 2 to 4 days or until the wound begins to close naturally.	Though widely trusted in the local system, the efficacy and safety of this remedy have not been scientifically established, and sterile handling is essential to avoid secondary infections.	42	0.57	42	0.57	
	8	*Saraca asoca* (Roxb.) W.J.de Wilde [RKABSP-20]	Ashok	Tree	Open land	Angiosperm	Root, leaves, bark	Paste	Ulcers (NIC-26), cancer (NIC-20), bone fracture (NIC-13)	Oral	In the Baiga healing system, a paste prepared from the root, leaves, and bark of this plant is consumed orally to address internal ailments such as ulcers, cancer, and to support bone healing in fractures. The fresh parts are ground together using a stone or wooden mortar, often with water or buttermilk, to make a thick paste. This is taken once daily, usually in the morning before meals, over a course of 10 to 15 days. The combination is considered potent in reducing internal inflammation and promoting regeneration.	However, no clinical trials support its efficacy, and unsupervised use—especially for serious conditions like cancer—is not advisable.	30	0.41	59	0.80	
	9	*Senegalia catechu* (L.f.) P.J.H.Hurter & Mabb. [RKABSP-36]	Khair	Tree	Open land	Angiosperm	Root, bark	Decoction	Piles (NIC-11), cancer (NIC-39)	Oral	The plant parts are boiled in water over low flame until the volume reduces significantly, yielding a concentrated preparation. This decoction is consumed orally, once daily, preferably on an empty stomach, for a period ranging from 7 to 15 days, depending on the condition. It is believed to relieve swelling and pain in piles and to purify the blood in cancer-related conditions.	While widely used among tribal communities, this remedy lacks empirical validation, and its internal use must be approached with caution, particularly in immunocompromised individuals.	39	0.53	50	0.68	
	10	*Senna hirsuta* (L.) H.S.Irwin & Barneby [RKABSP-40]	Kasam	Shrub	Road side	Angiosperm	Leaves	Infusion	Kidney stone (NIC-15)	Oral	Fresh leaves are soaked in lukewarm water overnight or steeped in hot water for several minutes to extract the active components. The strained liquid is consumed early in the morning, typically once a day, over a course of 5 to 10 days. This practice is believed to ease urinary flow and dissolve small stones.	While the remedy is popular among tribal healers, there is no scientific confirmation of its efficacy or safety, and excessive intake may affect renal function adversely.	15	0.20	15	0.20	
	11	*Vachellia nilotica* (L.) P.J.H.Hurter & Mabb. [RKABSP-79]	Babool	Tree	Open land	Angiosperm	Root	Decoction	Cancer (NIC-13)	Oral	Among the Baiga tribe, the root of this plant is boiled to prepare a decoction used in the management of cancer. The traditional method involves simmering crushed roots in water until the volume reduces, producing a dark, bitter liquid. This preparation is consumed once daily, generally in the early morning before food, and the course may extend from 15 to 21 days based on the advice of traditional healers. It is believed to purify the blood and arrest abnormal growths.	However, due to the serious nature of cancer and the absence of scientific validation, such remedies should not be used as substitutes for clinical treatment, especially without supervision.	13	0.18	13	0.18	
Lamiaceae	1	*Colebrookea oppositifolia* Sm. [RKABSP-11]	Ngarmotha, Pansre	Shrub	Forest	Angiosperm	Leaves	Paste	Cuts (NIC-12), asthma (NIC-08), dysentery (NIC-12)	External, Oral	For cuts, a fresh paste is prepared by crushing the leaves with clean water and applied directly over the affected area once or twice daily until healing is observed. For asthma and dysentery, the same paste is taken orally, typically once a day before meals for 4 to 7 days.	The remedy is valued for its cooling and anti-inflammatory properties. Despite its widespread traditional use, no pharmacological validation exists, and care should be taken to avoid overconsumption, particularly in individuals with digestive or respiratory sensitivity.	15	0.20	32	0.43	0.44
	2	*Gmelina arborea* Roxb. ex Sm. [RKABSP-41]	Gamber	Tree	Forest	Angiosperm	Leaves	Infusion	Cough (NIC-10)	Oral	To relieve cough, the Baiga tribe prepares an infusion from the leaves of this plant. Fresh or shade-dried leaves are steeped in warm water for several minutes to release their soothing compounds. The resulting liquid is filtered and consumed warm, typically once in the morning and once at night for 3 to 5 days.	Though widely practiced in tribal healthcare, its safety and efficacy have not been validated scientifically, and excessive intake is avoided due to the risk of gastrointestinal upset.	10	0.14	10	0.14	
	3	*Ocimum americanum* L. [RKABSP-23]	Tulsi	Shrub	Open land, road side	Angiosperm	Leaves	Infusion	Fever (NIC-43)	Oral	The Baiga community uses an infusion of the leaves of this plant to manage fever. Fresh leaves are steeped in hot water and allowed to infuse for some time, after which the liquid is strained and consumed warm. This herbal tea is generally taken twice a day morning and evening for 2 to 4 days, or until the fever subsides.	However, in the absence of clinical trials, this remedy should be used with care and not relied upon in severe or persistent fevers.	43	0.58	43	0.58	
	4	*Vitex negundo* L. [RKABSP-81]	Nirgundi	Shrub	Open land	Angiosperm	Bark	Paste	Menstrual cramps (NIC-30), mouth ulcer (NIC-16)	Oral	For preparation, the bark is pounded and mixed with a small amount of water to form a thick, fine paste. A small portion is consumed orally once daily, usually early morning or before bedtime. For menstrual discomfort, the treatment is continued for 2 to 3 days during the pain phase, while in cases of mouth ulcers, it is used for up to 5 days.	The remedy is believed to have cooling and analgesic effects. Despite its traditional popularity, there is no pharmacological validation, and its internal use is avoided during pregnancy or in individuals with known allergies to plant bark.	35	0.47	46	0.62	
Linaceae	1	*Linum usitatissimum* L. [RKABSP-17]	Tisi	Herb	Home garden	Angiosperm	Whole plant	Infusion	Cancer (NIC-18)	Oral	In Baiga traditional medicine, an infusion made from the whole plant is used orally to manage cancer-related symptoms. The aerial and underground parts are cleaned, chopped, and soaked in warm water to prepare a mild infusion. This preparation is consumed once daily, preferably early in the morning on an empty stomach, and continued for 10 to 15 days under the guidance of traditional healers. It is believed to cleanse the blood and slow the progression of abnormal tissue growth.	While this practice is deeply rooted in tribal knowledge, it lacks pharmacological validation and should be approached with caution, especially in individuals undergoing conventional cancer treatment.	18	0.24	18	0.24	0.24
Lythraceae	1	*Woodfordia fruticosa* (L.) Kurz [RKABSP-59]	Dhawai	Shrub	Forest, open land	Angiosperm	Root, leaves	Paste	Herpes (NIC-08), wounds (NIC-50)	External	The plant parts are pounded together with water or cow milk to form a fine paste, which is applied directly over the affected skin once or twice daily. For herpes, the application continues for 5 to 7 days to reduce itching, swelling, and blisters. In wound care, the paste is used until healing is visible.	However, no scientific studies currently confirm its efficacy or dermatological safety, and prolonged use on open wounds is discouraged.	50	0.68	58	0.78	0.78
Malvaceae	1	*Hibiscus × rosa-sinensis* L. [RKABSP-28]	Madaar	Shrub	Home garden	Angiosperm	Bark	Decoction	Heart problem (NIC-66)	Oral	Strips of bark are boiled in water over low flame until the volume reduces, producing a concentrated liquid. This decoction is consumed once daily, usually early in the morning, for a period of 7 to 10 days depending on the condition and the healer’s advice. The remedy is believed to strengthen cardiac function and regulate blood circulation.	While valued in folk medicine, this practice lacks clinical validation, and its use is approached cautiously, particularly among elderly individuals or those with diagnosed heart conditions.	66	0.89	66	0.89	0.89
Marsileaceae	1	*Marsilea quadrifolia* L. [RKABSP-72]	Sunasuniya	Herb	Open land	Pteridophyter	Leaves	Infusion	Epilepsy (NIC-11), piles (NIC-22)	Oral	Cleaned fresh or shade-dried leaves are soaked in lukewarm water and left to infuse overnight. The filtered liquid is then consumed early in the morning, typically once daily. For epilepsy, the remedy is continued for up to two weeks, while in the case of piles, it is administered for 5 to 7 days or until relief is observed.	It is believed to possess calming and anti-inflammatory effects. Although well accepted in folk medicine, scientific validation of this practice is still lacking, and use in children or those under medical treatment is approached with caution.	24	0.32	32	0.43	0.43
Meliaceae	1	*Azadirachta indica* A.Juss. [RKABSP-15]	Neem	Tree	Open land	Angiosperm	Leaves, bark	Paste	Skin infection (NIC-31), wound (NIC-41)	External	The fresh parts are ground on a stone slab using water until a thick paste forms. This is applied gently over the affected area, generally twice a day, in the morning and evening. For skin infections, it is continued for 4 to 6 days, while wounds are treated until the injury begins to dry and scar formation appears.	The preparation is believed to possess antimicrobial and healing properties. Despite its widespread local use, no toxicological data are available, so sensitive skin types are advised caution.	45	0.61	72	0.97	0.7
	2	*Melia azedarach* L. [RKABSP-56]	Bakain	Tree	Open land	Angiosperm	Bark	Paste	Piles (NIC-32)	Oral	Fresh bark is pounded into a fine paste using clean water or mixed with honey or buttermilk as per traditional knowledge. A small amount of this preparation is taken orally once a day, preferably early in the morning before food. The treatment is usually continued for 5 to 7 days, depending on the severity.	It is believed to help in reducing swelling and easing bowel movements. Although considered effective by local healers, no clinical studies exist to support its efficacy, and its internal use is discouraged for pregnant women and individuals with chronic digestive disorders.	32	0.43	32	0.43	
Menispermaceae	1	*Tinospora cordifolia* (Willd.) Hook.f. & Thomson [RKABSP-65]	Giloy	Climber	Open land, forest	Angiosperm	Stem	Decoction	Fever (NIC-30), jaundice (NIC-18)	Oral	Stem pieces are chopped and gently boiled in water until the liquid reduces to nearly half its original volume. This decoction is consumed once daily, either on an empty stomach in the morning or before bedtime. For fever, it is administered for 2 to 3 days, while for jaundice, the duration extends up to 7 to 10 days.	The remedy is traditionally believed to purify the blood and regulate body heat. However, its safety and hepatoprotective effects are yet to be validated through pharmacological studies.	36	0.49	48	0.65	0.64
Moraceae	1	*Ficus benghalensis* L. [RKABSP-21]	Barh, bargachh	Tree	Open land	Angiosperm	Bark, latex, leaves	Decoction, raw	Diabetes (NIC-27), wounds (NIC-14)	Oral, External	For diabetes, a decoction is prepared by boiling the bark and leaves together in water until reduced. This is taken orally, usually early in the morning, for a period ranging from 7 to 15 days depending on blood sugar symptoms. In the case of wounds, the raw latex or a freshly prepared leaf paste is applied directly to the affected area once or twice daily to promote healing and prevent infection.	The preparation is believed to have both blood-sugar-lowering and antimicrobial properties. Despite its frequent traditional use, caution is advised due to the potential toxicity of raw latex and lack of clinical evaluation.	31	0.42	41	0.55	0.46
	2	*Ficus semicordata* Miq. [RKABSP-33]	Dumar	Tree	Open land	Angiosperm	Bark, fruit	Paste	Leprocy (NIC-16), diarrhoea (NIC-34)	Oral	The plant parts are crushed together using a stone grinder, occasionally blended with a few drops of warm water or goat milk to ease ingestion. For leprosy, the paste is taken orally once a day over an extended period as advised by traditional healers. In cases of diarrhoea, it is consumed immediately after symptom onset and continued for 2 to 3 days.	The remedy is traditionally believed to purify the blood and regulate bowel movements. Internal use, however, is approached cautiously due to the absence of pharmacological validation and the potential presence of bioactive compounds that may cause side effects.	42	0.57	50	0.68	
	3	*Morus alba* L. [RKABSP-19]	Tut	Tree	Home garden	Angiosperm	Bark	Paste	Insomnia (NIC-12)	Oral	The fresh bark is ground into a fine paste and mixed with a small quantity of warm water or cow milk before bedtime. This remedy is taken orally once at night for 3 to 5 days, or until the symptoms subside. Local healers believe it calms the mind and promotes natural sleep without causing drowsiness during the day.	While widely accepted in traditional practice, the neuroactive properties of the plant remain unverified through scientific research, and its use is avoided in children, lactating mothers, and individuals with neurological conditions unless guided by experienced practitioners.	12	0.16	12	0.16	
Moringaceae	1	*Moringa oleifera* Lam. [RKABSP-50]	Munga	Tree	Home garden	Angiosperm	Bark	Paste	Diabetes (NIC-12), arthritis (NIC-10)	Oral	The bark is pounded into a paste and traditionally consumed once daily, preferably in the early morning on an empty stomach. For diabetes, the treatment may continue up to 10–15 days, depending on blood sugar response, while in arthritis, it is generally used for a shorter period (5–7 days) to reduce pain and stiffness. Sometimes it is taken along with warm water or mixed in buttermilk to improve taste and digestion.	Though culturally trusted for its anti-inflammatory and blood-glucose-regulating properties, its internal use remains unsupported by clinical validation and should be approached with care, especially by individuals under modern antidiabetic or anti-inflammatory medications.	15	0.20	22	0.30	0.3
Nyctaginaceae	1	*Boerhavia diffusa* L. [RKABSP-54]	Gadhapurna	Herb	Open land	Angiosperm	Root, leaves	Paste	Anemia (NIC-13), diabetes (NIC-22)	Oral	The plant parts are crushed together on a stone slab and the paste is taken once a day, usually in the morning before meals. For anaemia, it is often consumed with jaggery or mixed in goat milk to enhance blood-building effects, and the duration typically spans 7 to 10 days. In the case of diabetes, the paste is continued for a longer period as advised by local healers, sometimes along with dietary restrictions.	Though it is widely accepted by the community for boosting vitality and managing sugar levels, there is no pharmacological validation available, and care is advised in individuals with underlying health conditions or those already under medication.	26	0.35	35	0.47	0.55
	2	*Mirabilis jalapa* L. [RKABSP-58]	Gulabbas	Herb	Home garden	Angiosperm	Whole plant	Decoction	Inflammation (NIC-29), fever (NIC-17)	External	The plant is boiled in water over a low flame until the liquid reduces and becomes concentrated. This warm decoction is used to sponge the body, particularly around swollen joints and the forehead, once or twice daily. In some cases, a cloth is soaked in the decoction and wrapped around inflamed areas to reduce heat and swelling. This treatment is continued for 2 to 4 days depending on symptom severity.	While commonly practiced, the remedy lacks scientific validation, and its prolonged use is avoided in individuals with sensitive skin or open wounds.	34	0.46	46	0.62	
Oleaceae	1	*Jasminum sambac* (L.) Aiton [RKABSP-97]	Chameli, Mogra	Shrub	Home garden	Angiosperm	Flower	Infusion	Cough (NIC-17), anxiety (NIC-6)	Oral	Fresh or sun-dried flowers are steeped in warm water for a few minutes, and the infusion is consumed once or twice daily — usually early morning for anxiety and at bedtime or after meals in case of persistent cough. For both ailments, the practice is continued for 3 to 5 days or until the symptoms reduce. In anxiety, the flower tea is sometimes consumed with a pinch of jaggery or honey to enhance its soothing effect.	Although widely accepted in folk medicine, the remedy lacks scientific evidence regarding its safety and efficacy, and traditional healers advise against overuse.	17	0.23	23	0.31	0.28
	2	*Nyctanthes arbor-tristis* L. [RKABSP-62]	Parijat	Tree	Home garden	Angiosperm	Flower	Raw	Anti-fertility in female (NIC-19)	Oral	The flowers are chewed early in the morning for a few consecutive days, generally beginning after the menstrual cycle. This practice is believed to suppress ovulation or interfere with conception, serving as a temporary form of birth control. Women who do not wish to conceive rely on this remedy under the advice of elder women or local healers.	Despite its regular use in traditional settings, there is no scientific validation of its efficacy or safety. Therefore, use during pregnancy, lactation, or by women with reproductive health issues is strictly avoided.	19	0.26	19	0.26	
Oxalidaceae	1	*Biophytum sensitivum* (L.) DC. [RKABSP-94]	Lajwanti	Herb	Open land	Angiosperm	Whole plant	Infusion	Acidity (NIC-17), ulcer (NIC-20)	Oral	For preparation, the fresh or shade-dried plant is soaked in lukewarm water and left undisturbed for a few hours. The resulting infusion is consumed orally, typically twice a day—morning and evening—before meals to soothe gastric discomfort and promote healing of ulcers. The treatment is continued for about 3 to 5 days depending on severity.	Healers recommend a light diet during this period and advise avoiding sour or spicy foods. Though widely practiced, no clinical data supports its safety or long-term use, and caution is advised, particularly for individuals with chronic gastrointestinal disorders.	22	0.30	37	0.50	0.53
	2	*Oxalis corniculata* L. [RKABSP-68]	Teen Pattia	Herb	Open land	Angiosperm	Whole plant	Infusion	Abortifacient (NIC-42)	Oral	The plant is soaked in lukewarm water for several hours, and the resulting infusion is consumed early in the morning, typically on an empty stomach. This remedy is administered during the early stages of suspected pregnancy to induce abortion. Local healers strongly caution that it is used only under specific circumstances, often in cases of unwanted or risky pregnancies. The infusion is usually taken for one to three consecutive days, depending on physical response	As this practice carries serious health risks and lacks scientific validation, its use is considered highly unsafe without proper medical supervision. It is strictly contraindicated for general reproductive health use.	42	0.57	42	0.57	
Passifloraceae	1	*Turnera ulmifolia* L. [RKABSP-48]	Sangra	Herb	Home garden	Angiosperm	Whole plant	Infusion	Diarrhoea (NIC-7), constipation (NIC-10)	Oral	For diarrhoea, a small quantity of the infusion is taken once or twice a day after meals to regulate bowel movement. In contrast, for constipation, a slightly stronger infusion is consumed early in the morning on an empty stomach to ease stool passage. This flexible traditional remedy is typically used for 2 to 3 days under the guidance of experienced healers.	It is believed to have a balancing effect on gut function. However, due to the absence of pharmacological validation, prolonged or unsupervised use is discouraged, especially in children and the elderly.	12	0.16	17	0.23	0.23
Pedaliaceae	1	*Sesamum indicum* L. [RKABSP-38]	Til	Herb	Home garden	Angiosperm	Seed, root	Paste	Burns (NIC-07), hair growth (NIC-05)	Oral, External	For treating minor burns, the fresh paste is applied directly over the affected skin to cool the area and reduce inflammation. For hair growth, the paste is gently massaged onto the scalp and left for some time before rinsing. In some cases, a small amount may be taken orally, typically under strict supervision of traditional healers, to nourish the body from within and support hair regeneration, though external application remains more common. Treatment duration varies from 3 to 7 days.	No scientific safety data exists for oral use, hence it is not recommended for unsupervised or prolonged internal consumption.	10	0.14	12	0.16	0.16
Phyllanthaceae	1	*Phyllanthus emblica* L. [RKABSP-52]	Aamla	Tree	Forest	Angiosperm	Bark, fruit	Decoction	Liver problem (NIC-31), fever (NIC-12), wound (NIC-24)	Oral	The bark and fruit are boiled together in water until the volume reduces, and the decoction is consumed orally, usually once or twice daily, depending on the severity. For liver complaints, it is commonly taken in the early morning for up to five days. In fever cases, the remedy is used during symptomatic episodes to help bring down body temperature. When used for internal wound recovery, it is typically combined with a light diet and rest.	Local healers recommend this treatment be used cautiously and not prolonged beyond a week without guidance, due to the potential for liver strain and the absence of pharmacological validation.	41	0.55	67	0.91	0.91
Plantaginaceae	1	*Scoparia dulcis* L. [RKABSP-18]	Mithi patti	Herb	Open land, road side	Angiosperm	Whole plant	Infusion	Diabetes (NIC-15)	Oral	The plant is soaked overnight in lukewarm water, and the resulting liquid is consumed early in the morning on an empty stomach. This practice is usually followed for several consecutive days, often ranging from five to seven days, depending on the individual’s condition. The remedy is believed to help regulate blood sugar levels and is typically used under the guidance of experienced traditional healers.	However, as scientific validation of its efficacy and safety is lacking, this treatment is not recommended for unsupervised or long-term use, particularly in patients already on modern antidiabetic medication.	15	0.20	15	0.20	0.2
Poaceae	1	*Cynodon dactylon* (L.) Pers. [RKABSP-27]	Dhoob ghas	Herb	Open land	Angiosperm	Leaves, whole plant	Paste	Syphilis (NIC-17), diabetes (NIC-20), urine infection (NIC-12)	External, Oral	For syphilitic symptoms, the paste is applied externally over affected skin lesions, usually twice a day, until visible relief is observed. For managing diabetes and urinary infections, a small amount of the same paste is taken orally, generally on an empty stomach in the morning for five to seven days. It is often advised by local healers to avoid oily or spicy food during this period.	While this remedy remains widely practiced among traditional communities, its internal use is approached cautiously due to lack of pharmacological validation and potential for adverse effects if misused or overused.	31	0.42	49	0.66	0.66
Pteridaceae	1	*Adiantum philippense* L. [RKABSP-46]	Hansraj	Herb	Open land, forest	Pteridophyter	Whole plant	Paste	Lower cholesterol level (NIC-14)	Oral	The paste is usually taken orally, once daily in the early morning, often mixed with lukewarm water or taken plain, depending on the individual’s tolerance. The remedy is continued for a few consecutive days, typically not exceeding one week, and is usually accompanied by dietary advice such as avoiding fried or fatty foods. Local healers believe this preparation helps in cleansing the blood and improving metabolism.	However, due to the absence of clinical validation, the use of this remedy should be approached with caution, especially for individuals already on lipid-lowering medications.	14	0.19	14	0.19	0.19
Rhamnaceae	1	*Ziziphus jujuba* Mill. [RKABSP-95]	Ber	Tree	Open land, forest	Angiosperm	Root	Decoction	Constipation (NIC-09)	Oral	The root is boiled in water until the liquid reduces to nearly half, then strained and consumed warm, preferably at night before sleeping. This practice is followed for one to three days, depending on the severity of the condition. The remedy is said to promote smooth bowel movement and is often accompanied by increased water intake and a light, fiber-rich diet.	Traditional healers advise not to exceed the recommended duration, as excessive use may lead to weakness or dependency. No scientific validation currently exists to support its long-term safety.	9	0.12	9	0.12	0.12
Rubiaceae	1	*Ixora coccinea* L. [RKABSP-24]	Rukmini	Shrub	Home garden	Angiosperm	Root	Decoction	Leucorrhoea (NIC-04), dysentery (NIC-09)	Oral	A decoction is prepared by boiling the root in water until reduced to half, then filtered and taken orally. For leucorrhoea, it is usually administered early in the morning, often for five to seven consecutive days, with dietary restrictions such as avoiding sour and oily food. In the case of dysentery, the same decoction is consumed two to three times a day, depending on the severity, until symptoms subside.	Tribal healers recommend its use under supervision and discourage prolonged intake due to the lack of pharmacological studies supporting its safety profile.	10	0.14	13	0.18	0.2
	2	*Neolamarckia cadamba* (Roxb.) Bosser [RKABSP-29]	Kadam	Tree	Open land	Angiosperm	Bark	Decoction	Cancer (NIC-17)	Oral	The bark is boiled in water until the volume is reduced, then strained and consumed orally once or twice a day. The decoction is usually taken on an empty stomach in the morning or before meals for a duration ranging from one week to fifteen days, depending on the condition and the healer’s guidance. While considered a supportive remedy to reduce internal inflammation and tumor-associated pain, it is administered cautiously, especially in elderly or weak patients.	Traditional practitioners often advise avoiding meat, salt, and spicy foods during this treatment period. However, its efficacy and safety remain unvalidated by scientific studies, and prolonged or unsupervised use is discouraged.	17	0.23	17	0.23	
Rutaceae	1	*Aegle marmelos* (L.) Corrêa [RKABSP-98]	Bel	Tree	Open land, home garden	Angiosperm	Leaves, bark, fruit	Infusion, decoction	Nausea (NIC-08), dyspepsia (NIC-07), cancer (NIC-27), blood pressure (NIC-14)	Oral	For nausea and dyspepsia, a mild infusion is prepared and consumed orally two to three times daily after meals to aid digestion and reduce discomfort. In the case of cancer and blood pressure-related issues, a stronger decoction is preferred, usually taken early in the morning and sometimes in the evening, as advised by local healers. The treatment is continued for several days up to two weeks, often with dietary restrictions such as avoiding spicy or fried foods.	Though widely practiced in the community, this traditional remedy lacks clinical evidence and should not replace conventional treatment. Caution is advised, especially in pregnant women and individuals on antihypertensive medications.	36	0.49	56	0.76	0.78
	2	*Citrus × limon* (L.) Osbeck [RKABSP-53]	Neembu	Tree	Home garden	Angiosperm	Fruit	Juice	Menstrual pain (NIC-59)	Oral	Women consume a small amount of the juice generally extracted by manually crushing the pulp and filtering it once daily during the initial days of the menstrual cycle when cramps are most intense. It is typically taken in the early morning on an empty stomach or before meals for 2 to 3 consecutive days.	The remedy is believed to ease uterine contractions and reduce abdominal discomfort. Traditional healers may also recommend avoiding sour foods and cold water during treatment. While the practice is culturally significant and widely followed, the pharmacological basis of this remedy remains undocumented, and caution is advised, especially in individuals with known uterine or hormonal conditions.	59	0.80	59	0.80	
Sapotaceae	1	*Madhuca longifolia* var. latifolia (Roxb.) A.Chev. [RKABSP-67]	Mohwa	Tree	Open land, forest	Angiosperm	Bark, root	Decoction	Cancer (NIC-34), ulcer (NIC-20)	Oral	The ingredients are boiled together in water until the liquid is reduced to approximately half, then allowed to cool and strained. This decoction is consumed orally, usually once in the morning and, in some cases, again in the evening, particularly for chronic ulcers or pain-related cancer symptoms. The treatment duration typically ranges from 7 to 15 days under the supervision of traditional healers.	It is believed to reduce internal inflammation, soothe ulcerative pain, and support overall well-being. During treatment, patients are often advised to follow dietary restrictions such as avoiding sour, spicy, and non-vegetarian foods. Despite its cultural importance, scientific validation of its efficacy is lacking, and internal use should be approached with caution in individuals with hepatic or renal disorders.	39	0.53	54	0.73	0.73
Solanaceae	1	*Datura metel* L. [RKABSP-78]	Safed datura	Shrub	Open land	Angiosperm	Root	Decoction	Arthritis (NIC-45)	External	The root is boiled in water for an extended time until the liquid becomes moderately concentrated. After cooling to a tolerable temperature, the decoction is gently massaged over the affected joints or used as a warm compress, typically twice daily morning and evening for a period of 7 to 10 days. This remedy is believed to improve joint mobility and reduce swelling.	Local healers often advise patients to avoid cold exposure and to rest the joints during treatment. Though widely used in traditional settings, this practice lacks clinical validation and should be used with care, especially in individuals with sensitive skin or open wounds.	45	0.61	45	0.61	0.67
	2	*Nicotiana tabacum* L. [RKABSP-83]	Tambaku	Herb	Home garden	Angiosperm	Leaves, root	Infusion	Headaches (NIC-17), cancer (NIC-20)	Oral	Fresh or shade-dried plant parts are soaked in lukewarm water overnight or for several hours, then filtered and taken orally, preferably in the early morning on an empty stomach. For headaches, the infusion is usually administered for 2 to 3 days until relief is achieved. In cancer cases, it is given daily for up to two weeks under the guidance of traditional healers.	During treatment, the intake of spicy, oily, and heavy food is generally discouraged. While culturally significant, there is no scientific validation for its efficacy, and long-term internal use should be approached with caution.	32	0.43	37	0.50	
	3	*Withania somnifera* (L.) Dunal [RKABSP-80]	Ashwagandha	Shrub	Forest	Angiosperm	Root	Paste	Rheumatism (NIC17-), cuts (NIC-49)	External	For rheumatic pain, freshly pounded root is applied warm over affected joints and muscles, generally twice a day—morning and evening—for about 5 to 7 days. It is often covered with a cloth to retain warmth and promote better absorption. For cuts and skin injuries, the paste is applied directly to the wound site to stop bleeding, prevent infection, and accelerate healing.	Healers caution against exposure to cold air or water during the treatment. While widely practiced, the remedy lacks modern clinical validation, and potential allergic reactions from topical use should be considered.	52	0.70	66	0.89	
Zingiberaceae	1	*Curcuma amada* Roxb. [RKABSP-16]	Jungli haldi	Herb	Forest	Angiosperm	Rhizome	Paste	Asthma (NIC-39)	Oral	The rhizome is cleaned, crushed into a fine paste, and traditionally consumed once daily in the early morning, usually mixed with a small quantity of warm water or honey to mask bitterness. The treatment is continued for about 5 to 7 days or until breathing difficulty subsides. It is believed to help clear mucus, ease bronchial constriction, and strengthen respiratory function.	Patients are generally advised to avoid cold food and drinks during this period. Despite its cultural acceptance, the remedy has not been scientifically validated for dosage, efficacy, or safety, especially in individuals with chronic respiratory disorders.	39	0.53	39	0.53	0.54
	2	*Curcuma angustifolia* Roxb. [RKABSP-86]	Tikhur	Herb	Forest	Angiosperm	Rhizome	Decoction	Bronchitis (NIC-23)	Oral	The rhizome is sliced or pounded and then boiled in water until the volume reduces by half. This decoction is allowed to cool slightly and consumed warm once or twice daily, preferably on an empty stomach or before bedtime, for 3 to 5 days. It is believed to help clear phlegm, ease throat irritation, and reduce chest congestion.	During this treatment, individuals are usually advised to avoid cold food, curd, or exposure to dust and cold wind. Although widely practiced, the remedy lacks scientific validation, and care should be taken in patients with chronic respiratory or gastrointestinal sensitivity.	23	0.31	23	0.31	
	3	*Zingiber officinale* Roscoe [RKABSP-89]	Adrak	Herb	Home garden	Angiosperm	Rhizome	Paste	Nausea (NIC-30), headaches (NIC-27)	Oral	A small amount of freshly ground rhizome paste is traditionally taken once in the morning, often mixed with honey or lukewarm water to ease the bitter taste. For nausea, it is given as soon as symptoms appear, usually continued for 2 to 3 days. In the case of headaches, the remedy is consumed for up to 5 days, sometimes accompanied by rest in a quiet, shaded area.	Elders advise avoiding oily or spicy food during treatment. While this remedy is widely practiced in local traditions, there is a lack of scientific validation regarding dosage safety and active compounds.	42	0.57	57	0.77	

Abbreviation: NIC = number of informants cited, UV = use value, FUV = family use value, FC = frequency citation, RFC = relative frequency citation.

Families represented by two species, including Anacardiaceae, Apocynaceae, Asteraceae, and Cucurbitaceae, indicate moderate representation. These families contribute to the structural complexity of vegetation and serve as critical food sources for various fauna. Notably, families represented by a single species, such as Acanthaceae, Apiaceae, Dipterocarpaceae, and Rhamnaceae, signify the presence of rare or niche-specific taxa. These single-species families may represent taxa with restricted distributions, specialized ecological functions, or sensitivity to environmental changes. Their presence underscores the conservation value of the study area and highlights the need for biodiversity preservation efforts.

### Habit and habitat of documented medicinal plants

**Fig. 4:**
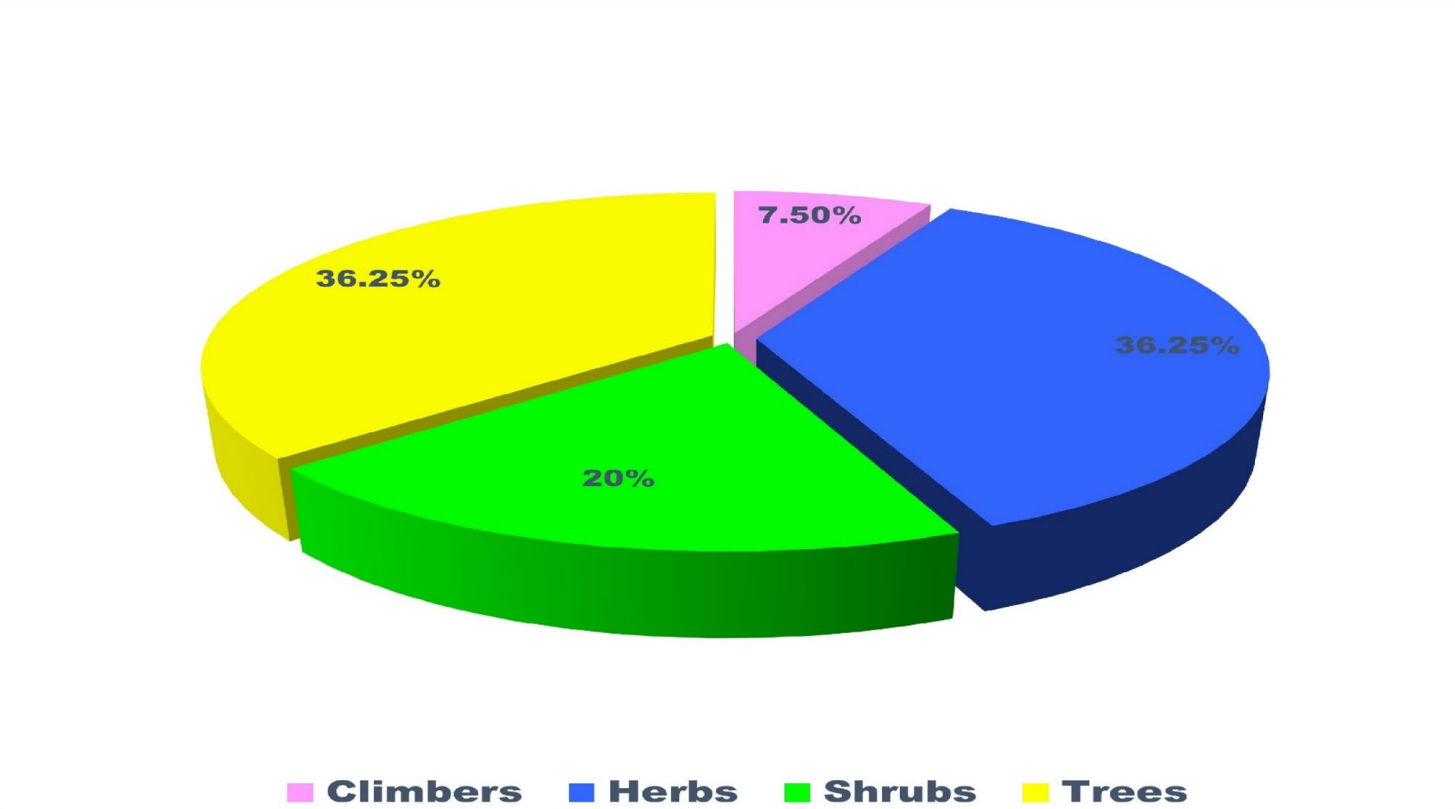
Habit of the medicinal plants.

The results of this study underscore the crucial role of plant habits in the realm of ethnobotanical applications. A striking 36% of the species identified belong to both herbs and trees, reflecting their pivotal roles in traditional medicine, food, and cultural practices. In Ethiopia, herbs emerged as the dominant habit for medicinal plants^2^. Valued for their medicinal properties, herbs are often the foundation of traditional healthcare systems, thanks to their bioactive compounds and ease of accessibility. Trees, conversely, are indispensable for providing timber, fruits, and various ethnobotanical uses, such as shade and religious purposes in rural and indigenous communities^51^. Shrubs, representing 20% of the species (Fig. 4), serve purposes ranging from medicinal and ornamental to forage, while climbers, though fewer in number (7%), hold their own significance, providing materials for ropes, traditional construction, and remedies for a variety of ailments^52^. This diverse array of plant habits highlights the broad spectrum of uses within the study area, fulfilling both subsistence and cultural needs.

**Fig. 5:**
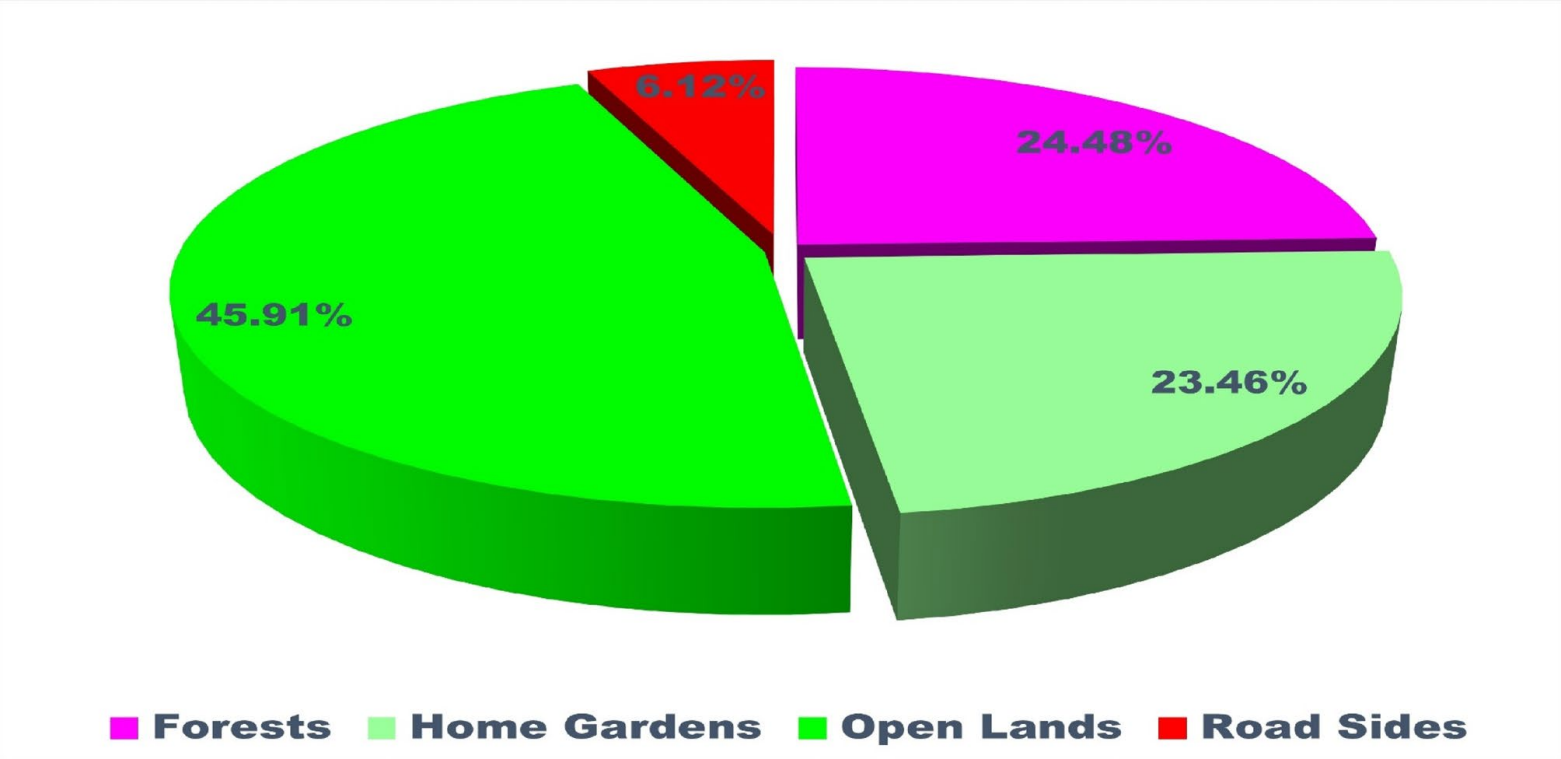
Habitat of the medicinal plants.

The habitat analysis reveals the wide-reaching ethnobotanical significance of species across varying landscapes. Open lands, supporting the highest proportion of species (45%), are essential gathering grounds for medicinal plants, wild edibles, and other valuable species (Fig. 5). These areas are vital for providing easy access to plant resources, playing a central role in preserving traditional practices. Forests, comprising 24% of the species, are treasure troves of ethnobotanical resources, offering plants used in traditional medicine, food, and rituals. These spaces are foundational to indigenous knowledge systems^53^. Similarly, home gardens, which house 23% of the species, serve as curated spaces for cultivating culturally significant plants. They act as living repositories of traditional wisdom, nurturing medicinal herbs, fruit-bearing trees, and ornamental plants. Roadside habitats, despite representing a smaller portion (6%), still demonstrate their ethnobotanical relevance. Plants found along roadsides are often opportunistically used for fodder, fuel, or as quick remedies in rural communities.

### Parts of plants used in the treatment of human ailments

**Fig. 6:**
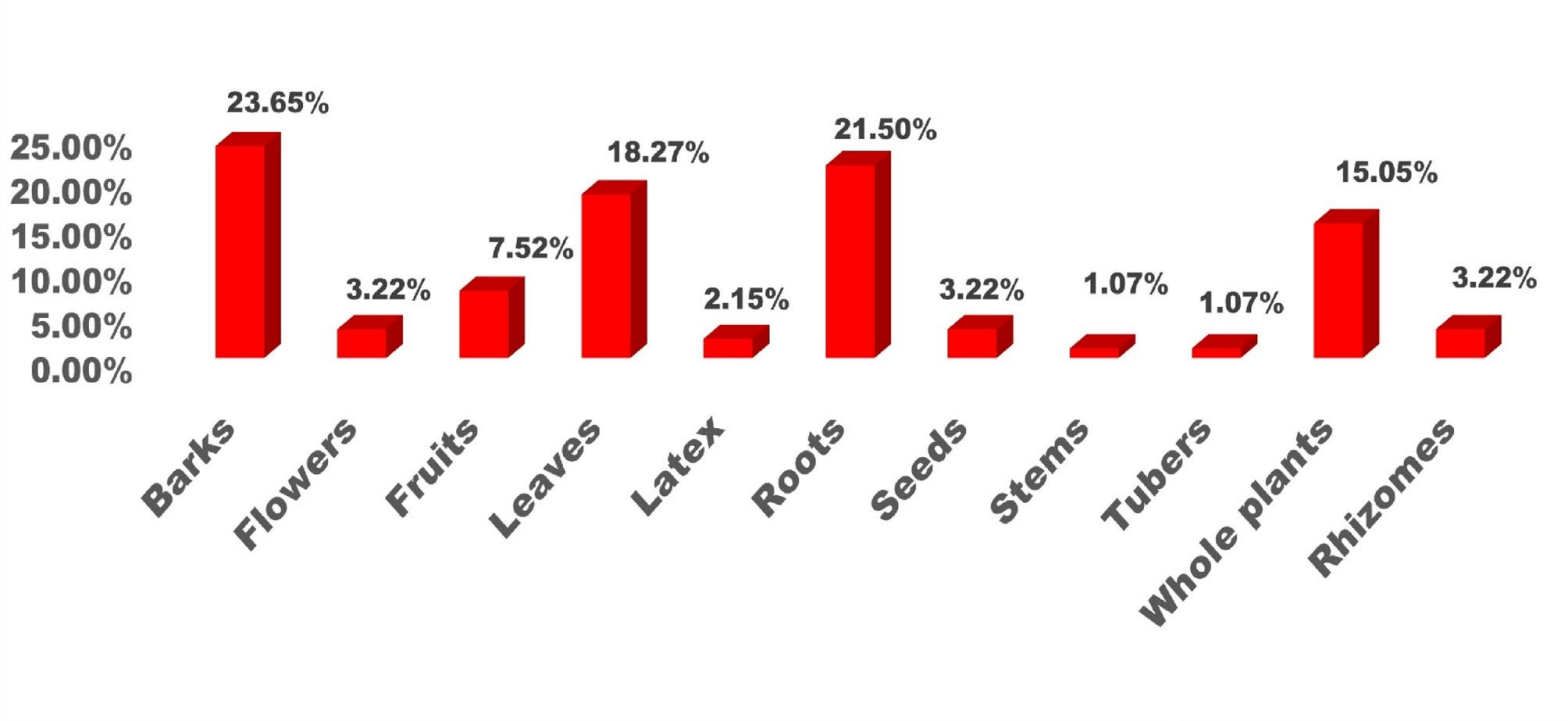
Plant parts used in human ailments treatments.

The Baiga tribe’s traditional knowledge reflects a profound understanding of the healing power of plants, showcasing their intimate relationship with nature. Among the various plant parts utilized in treating human ailments, barks stand out as the most frequently used (Fig. 6), accounting for 23%. This significant reliance on bark extracts is a testament to their effectiveness in addressing a range of conditions, including fevers, inflammations, and infections^54^. Enriched with bioactive compounds like tannins and alkaloids, barks are a cornerstone of the Baiga tribe’s herbal pharmacopoeia^55^. Roots, contributing 21%, hold a place of vital importance in their healing practices. Through decoctions and infusions, roots are employed to combat gastrointestinal disorders, respiratory issues, and systemic ailments. Their widespread use underscores their deep cultural and medicinal value, reflecting the tribe’s belief in their potent curative properties. Leaves, making up 18%, are indispensable in the Baiga tribe’s herbal remedies. Valued for their anti-inflammatory, antiseptic, and wound-healing abilities, they are often applied as teas, poultices, or topical treatments^56^. The holistic utilization of whole plants (15%) highlights the tribe’s approach to harnessing the full spectrum of a plant’s medicinal potential, ensuring no part goes to waste. Fruits (7%) play a dual role, offering both nutritional and therapeutic benefits. They are particularly useful in managing digestive and metabolic issues^57^. The delicate and purposeful use of flowers (3%) and seeds (3%), often in treatments for skin conditions and reproductive health, further emphasizes the diversity of the Baiga tribe’s ethnobotanical repertoire. Rhizomes (3%), with their aromatic and bioactive properties, are prized for their ability to address inflammation and digestive disorders^58,59^. Less commonly utilized plant parts, such as latex (2%), are applied sparingly for their antiseptic and purgative effects, while stems (1%) and tubers (1%) are reserved for specialized remedies targeting specific ailments. These practices illustrate the nuanced understanding of plant resources within the tribe, showcasing their ability to identify and utilize even the rarest plant parts for maximum benefit.

### Mode of preparation and administration of medicinal plants

**Fig. 7:**
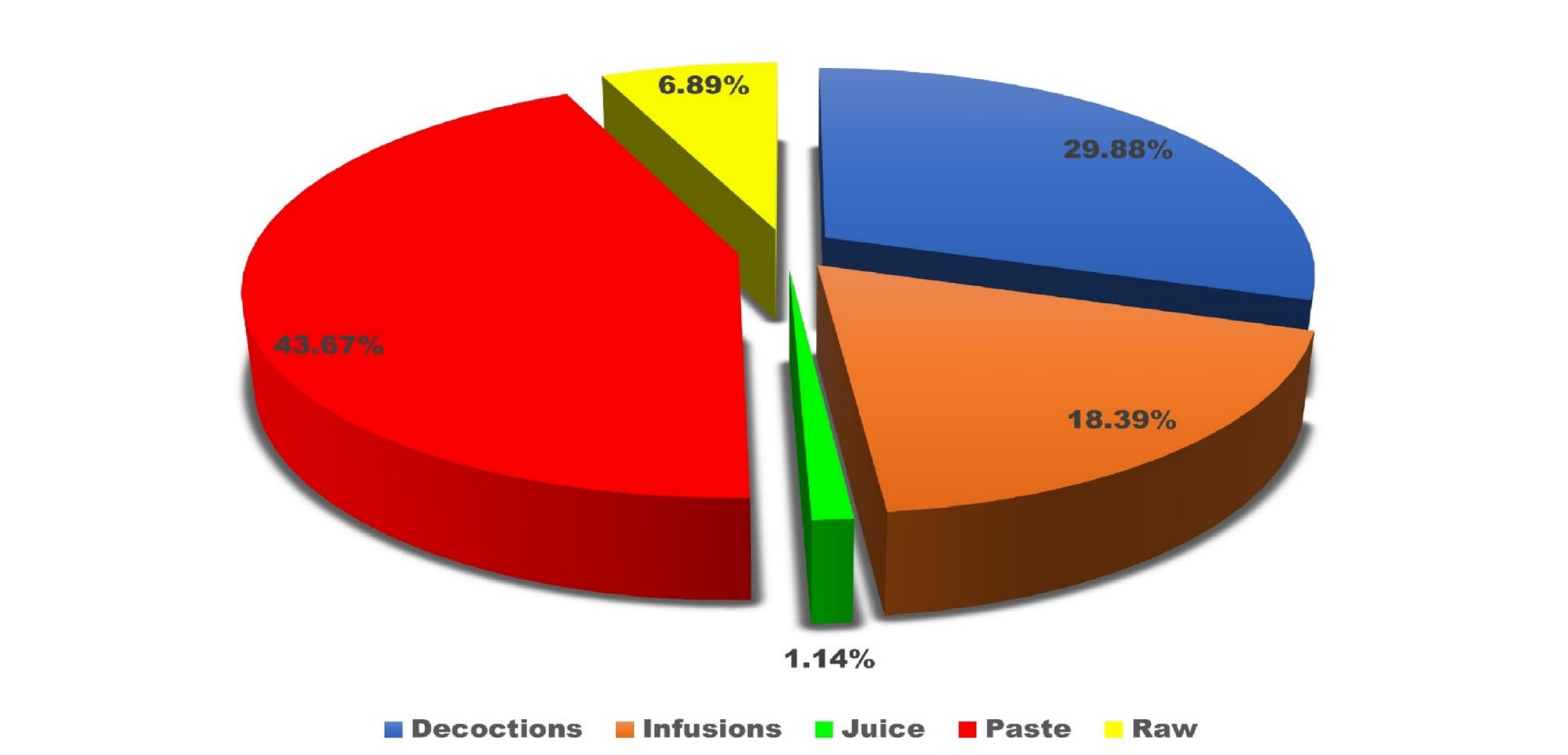
Mode of preparation of medicinal plants.

Among the various preparation methods, paste emerged as the most commonly used, accounting for 43% of the total reported preparations (Fig. 7). This high percentage suggests that the paste form is favoured for its ease of application and potent therapeutic properties. Often associated with a more concentrated preparation, paste may enhance effectiveness, especially for external use. Decoctions followed closely, representing 29% of cases. This preparation method, which involves boiling plant material in water, is prized for its ability to extract active compounds efficiently, making it a popular choice for medicinal purposes^60^. The widespread use of decoctions reflects a preference for strong extracts of bioactive compounds, believed to contribute significantly to the therapeutic benefits of the substance^61^. Infusions, making up 18% of cases, were used moderately compared to other preparations. Typically prepared by steeping plant material in hot water, infusions offer a gentler extraction process, preferred for substances that do not require intense heat. This moderate use suggests a balance between potency and gentleness in preparation. In contrast, juice (1%) and raw (6%) forms were less commonly used, indicating that these methods may either be less effective or culturally less significant for the intended purposes. The low preference for juice suggests that either the active compounds are less concentrated in this form, or that other preparation methods are perceived as more efficacious.

**Fig. 8:**
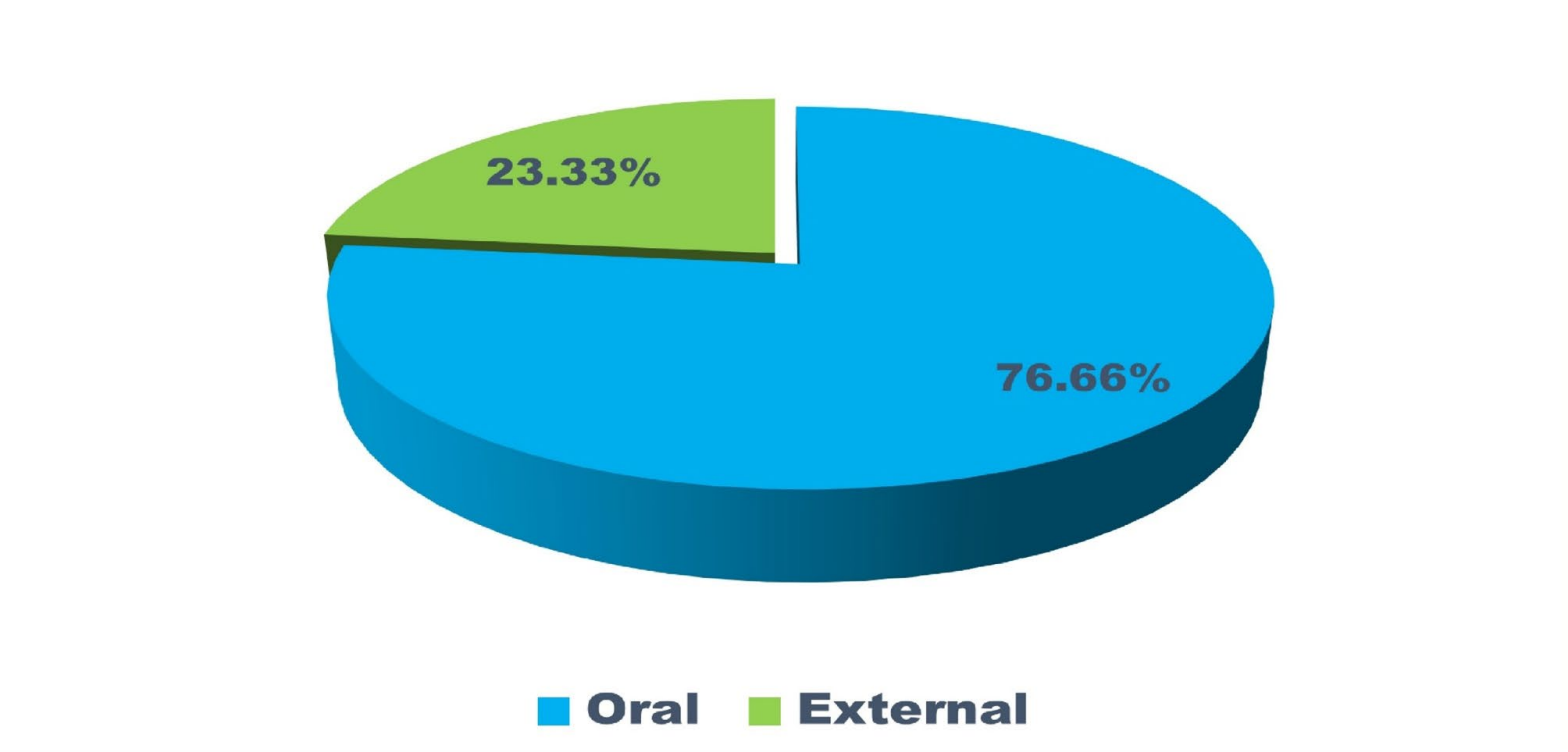
Mode of administration of medicinal plants.

In terms of administration, most cases (76%) involved oral consumption of the substance (Fig. 8). Similar findings were reported by^7^ and^62^. This highlights the importance of oral administration in traditional remedies, likely due to its systemic effects when ingested. Oral use is typically preferred for addressing internal health conditions, reflecting a widespread belief in the effectiveness of oral treatments. In contrast, external administration accounted for 23% of cases. While external applications are significant, especially for treating localized conditions or providing topical benefits, oral administration remains the dominant method.

### Use value (UV) and family use value (FUV)

The current study delves into the use value and family use value of plant species within the Baiga tribe’s traditional knowledge systems, uncovering fascinating insights into their cultural, medicinal, and economic practices. The findings reveal significant variations in the utility of plant species, with certain taxa emerging as pillars of the community’s livelihood and identity, while others hold lesser significance.

Among the assessed species, *Azadirachta indica* A.Juss., *Cyperus rotundus* L., *Hibiscus × rosa-sinensis* L., *Shorea robusta* C.F.Gaertn., and *Withania somnifera* (L.) Dunal stand out with remarkably high use values, underlining their indispensable roles in the Baiga way of life. Neem (*Azadirachta indica* A.Juss.) tops the list with an impressive score of 0.97, celebrated for its extraordinary versatility (Table 2). From traditional medicine and pest control to household products, neem’s widespread applications make it a cornerstone of local practices. Numerous studies corroborate its medicinal efficacy, highlighting its global significance^63–65^. *Shorea robusta* C.F.Gaertn. (sal), scoring 0.95, is deeply intertwined with the cultural and economic fabric of the Baiga tribe. As a source of timber, medicinal remedies, and an integral part of religious rituals, sal exemplifies the multifaceted utility of plants in sustaining both livelihood and traditions^66,67^. *Cyperus rotundus* L (0.91) and *Withania somnifera* (L.) Dunal (ashwagandha, 0.89) further emphasize the tribe’s reliance on medicinal plants. *Cyperus rotundus* L. is renowned for its broad therapeutic applications in traditional medicine, while ashwagandha, an adaptogenic herb, plays a crucial role in stress management and overall wellness^68^. *Hibiscus × rosa-sinensis* L. (0.89), known for its dual ornamental and medicinal value, underscores the aesthetic and practical dimensions of plant utility in the Baiga community^69^. Conversely, species like *Jatropha curcas* L. (0.08), *Colocasia esculenta* (L.) Schott (0.12), and *Ziziphus jujuba* Mill. (0.12) were identified as having low use values. Despite its potential as a biofuel crop, *Jatropha curcas* L. holds limited traditional significance, likely due to its relatively recent introduction and specialized applications^70^. *Colocasia esculenta* (L.), a staple food in other regions, appears to have minimal importance for the Baiga tribe, perhaps reflecting dietary preferences and the availability of alternative food sources^71^. Similarly, *Ziziphus jujuba* Mill., while recognized for its medicinal benefits, lacks the versatility that characterizes higher-ranking species such as *Azadirachta indica* A.Juss., and *Shorea robusta* C.F.Gaertn.

The evaluation of family use value revealed that certain plant families are particularly important within the Baiga tribe’s traditional knowledge systems. The Dipterocarpaceae family, with a score of 0.95, is exemplified by *Shorea robusta* C.F.Gaertn., a species that has significant cultural, medicinal, and economic value. The Cyperaceae family, represented by *Cyperus rotundus* L. (0.91), also ranks highly due to its wide range of uses in traditional medicine and its economic importance. Additionally, the Malvaceae family, with a score of 0.89, is represented by *Hibiscus × rosa-sinensis* L., highlighting the importance of ornamental and medicinal plants within this family. In contrast, the families Araceae (0.12) and Rhamnaceae (0.12) exhibited low family use values, which suggests that the species within these families are of limited practical importance in the context of the Baiga tribe’s traditional knowledge. This may be due to the specialized nature of the species within these families or the limited scope of their applications in the tribe’s daily life.

### Relative frequency citation (RFC)

In the present study, RFC values ranged from 0.08 to 0.89, reflecting considerable variability in the knowledge and use of medicinal plants among the studied population. Among the documented species, *Hibiscus × rosa-sinensis* L. exhibited the highest RFC value (0.89), indicating its widespread recognition and frequent use among the informants. Its high value can be attributed to its multiple therapeutic applications, such as in the treatment of skin infections, fever, and menstrual disorders. Moreover, the plant is commonly cultivated in household gardens, which increases its accessibility and familiarity^72^.

Following closely, *Dalbergia latifolia* Roxb. (RFC: 0.81), Momordica dioica Roxb. ex Willd. (0.80), and *Citrus × limon* (L.) Osbeck (0.80) were also frequently cited by the local people. The high RFC for Dalbergia latifolia Roxb. may be due to its recognized anti-inflammatory and analgesic properties, along with its traditional use in bone-related ailments. *Momordica dioica* Roxb. ex Willd. is widely used for digestive problems, infections, and as a nutritional food, which likely explains its popularity. Similarly, *Citrus × limon* (L.) Osbeck is well-known for its antimicrobial and digestive benefits and is a common household remedy, which enhances its cultural salience^73^.

In contrast, species like *Jatropha curcas* L. (RFC: 0.08) and *Colocasia esculenta* (L.) Schott (RFC: 0.09) showed the lowest RFC values. The limited use of *Jatropha curcas* L. may stem from its known toxicity and restricted therapeutic use, which discourages frequent mention or application in local healthcare. Meanwhile, *Colocasia esculenta* (L.) Schott, although primarily used as a food plant, may not hold significant medicinal relevance in the region, resulting in its lower citation frequency^63^.

Overall, these results suggest that plants with broad therapeutic applications, easy availability, and cultural familiarity tend to have higher RFC values. Conversely, those with limited medicinal uses, potential toxicity, or lesser recognition among the local communities score lower on this index. The RFC analysis, therefore, provides meaningful insight into the relative ethnobotanical value of each species and highlights the species most culturally and medicinally significant to the community.

### Informant consensus factor (ICF)

The informant consensus factor (ICF) values for various ailments were calculated to assess the level of agreement among informants regarding the use of medicinal plants for specific conditions. The highest ICF value of 0.97 was observed for both musculoskeletal and connective tissue disorders, such as bone fractures, arthritis, and menstrual cramps, and circulatory system conditions, such as anaemia and heart problems, reflecting a very strong and consistent knowledge base among informants regarding these ailments (Table 3). Close behind, skin and subcutaneous conditions, including wound healing, itching, and skin infections, had an ICF value of 0.96, indicating a high level of consensus regarding the plants used for these ailments. The ICF for infectious diseases was similarly high (0.96), emphasizing the consistent use of specific plants for conditions like tuberculosis and scabies. Endocrine and metabolic disorders, including diabetes, hypertension, and liver problems, also showed an ICF of 0.96, reflecting widespread agreement on the plants used for these critical health issues. The ICF for digestive system ailments, including constipation, diarrhoea, and dyspepsia, was 0.94, highlighting a strong but slightly less unanimous consensus among informants regarding the use of plants for gastrointestinal problems. The “Other General Conditions” category, which includes a variety of ailments such as cancer, fever, and insect bites, had an ICF of 0.94, indicating a high level of consensus but with some variation in the plant species used. Reproductive and urinary system disorders, with an ICF of 0.93, showed moderate agreement, suggesting some variability in the traditional use of plants for conditions like urinary tract infections and kidney stones. Lastly, neurological, and mental disorders, such as epilepsy and insomnia, had the lowest ICF of 0.92, suggesting a slightly lower consensus, possibly due to the complexity and variability of these conditions. Overall, the ICF values reveal that the community has a strong, consistent body of knowledge regarding the use of medicinal plants, especially for common and critical health issues, although there is slightly more variation for certain categories, particularly neurological and reproductive conditions.

**Table 3:** Informant consensus factor (ICF) values for different ailment categories, indicating the level of agreement among participants regarding the medicinal use of plants in the study area.

S. No	Ailments category	Ailments Name	No. of taxa used	Total number of informants cited	ICF
1	Respiratory System	Pneumonia, Bronchitis, Asthma, Coryza, Influenza, Cough	14	213	0.93
2	Digestive System	Constipation, Diarrhoea, Pyrosis, Dyspepsia, Dysentery, Ulcer, Nausea	17	312	0.94
3	Endocrine and Metabolic Disorders	Diabetes, Liver problem, Jaundice, Hypertension, Lower cholesterol level	18	432	0.96
4	Neurological and Mental Disorders	Epilepsy, Neuron problem, Insomnia, Anxiety	6	71	0.92
5	Infectious Diseases	Tuberculosis, Scabies, Dengue fever, Leprosy, Syphilis	6	131	0.96
6	Skin and Subcutaneous Conditions	Wound healing, Itching, Skin infection, Sores, Wound healing, Cuts	9	217	0.96
7	Musculoskeletal and Connective Tissue Disorders	Bone fracture, Menstrual cramps, Arthritis, Menstrual pain, Rheumatism	7	206	0.97
8	Reproductive and Urinary Systems	Kidney stone, Bladder catarrh, Urinary tract infection, Kidney stone, Leucorrhoea	5	61	0.93
9	Circulatory System	Anaemia, Heart problem, Blood pressure	4	101	0.97
10	Other General Conditions	Cancer, Tympanitis, Heat strokes, Swollen, Insect bite, Females problem, Leukoderma, Gastric ulcer, Febrifuge, Worms, Indigestion, Hepatitis, Anorexia, Inflammation, Ear pain, Gonorrhoea, Fever	20	346	0.94

### Population density patterns of ethnobotanically important tree species

The population density of tree species provides insight into their ecological distribution and availability, which often correlates with their ethnobotanical importance within indigenous communities. In the present study, density patterns were used to interpret the ecological standing of individual species and their relationship to traditional knowledge and usage among the Baiga tribe (Table 4).

**Table 4:** Importance value index (IVI) of documented medicinal plant species, reflecting their ecological dominance and ethnobotanical significance in the study area.

S.No.	Species	Local name	Family	Relative Density	Relative Frequency	Relative Dominance	Importance Value Index (I.V.I.)
1	*Aegle marmelos* (L.) Corrêa	Bel	Rutaceae	5.58	6.67	2.19	14.44
2	*Azadirachta indica* A.Juss.	Neem	Meliaceae	5.20	6.67	2.42	14.29
3	*Bauhinia variegata* L.	Kachnar	Fabaceae	5.95	5.00	2.19	13.14
4	*Butea monosperma* (Lam.) Kuntze	Palash	Fabaceae	5.20	5.00	2.19	12.40
5	*Cordia dichotoma* G.Forst.	Bakain	Boraginaceae	2.23	1.67	2.19	6.09
6	*Cordia macleodii* (Griff.) Hook.f. & Thomson	Dahiman	Boraginaceae	0.74	3.33	3.43	7.51
7	*Dalbergia latifolia* Roxb.	Sheesham	Fabaceae	2.97	3.33	11.11	17.42
8	*Diospyros melanoxylon* Roxb.	Tendu	Ebenaceae	9.29	8.33	27.26	44.89
9	*Ficus benghalensis* L.	Bargad	Moraceae	5.95	3.33	4.94	14.22
10	*Ficus semicordata* Miq.	Dumar	Moraceae	6.32	3.33	2.78	12.43
11	*Gmelina arborea* Roxb. ex Sm.	Khamhar	Lamiaceae	3.72	3.33	1.23	8.29
12	*Madhuca longifolia* var. latifolia (Roxb.) A.Chev.	Mohuwa	Sapotaceae	6.69	5.00	2.19	13.89
13	*Melia azedarach* L.	Bakain	Meliaceae	2.23	5.00	1.23	8.46
14	*Phyllanthus emblica* L.	Aamla	Phyllanthaceae	5.20	5.00	1.23	11.44
15	*Saraca asoca* (Roxb.) W.J.de Wilde	Ashok	Fabaceae	1.49	1.67	1.68	4.83
16	*Semecarpus anacardium* L.f.	Bhelwa	Anacardiaceae	6.69	5.00	2.19	13.89
17	*Senegalia catechu* (L.f.) P.J.H.Hurter & Mabb.	Khair	Fabaceae	2.97	3.33	2.19	8.50
18	*Shorea robusta* C.F.Gaertn.	Sal	Dipterocarpaceae	7.81	6.67	11.86	26.33
19	*Terminalia bellirica* (Gaertn.) Roxb.	Bahera	Combretaceae	4.09	5.00	6.72	15.81
20	*Terminalia chebula* Retz.	Harra	Combretaceae	2.97	3.33	3.43	9.74
21	*Vachellia nilotica* (L.) P.J.H.Hurter & Mabb.	Babool	Fabaceae	3.72	5.00	2.54	11.25
22	*Ziziphus jujuba* Mill.	Ber	Rhamnaceae	2.97	5.00	2.78	10.75

*Diospyros melanoxylon* Roxb. (Tendu) recorded the highest relative density (9.29%), suggesting its wide natural distribution and ecological dominance in the study area. This species is of great cultural and economic value to the Baiga community, particularly due to its leaves, which are extensively used in local bidi (traditional cigarette) production. Its fruits and bark are also used medicinally, contributing to its strong presence in both ecological and ethnobotanical domains^74,75^.

*Madhuca longifolia* var. latifolia (Roxb.) A.Chev. (Mohua) and *Semecarpus anacardium* L.f. (Bhelwa) both exhibited a relative density of 6.69%, underscoring their prominent ecological presence. Mohua is especially significant as its flowers are fermented to produce traditional alcohol, and its seeds yield oil used for cooking and skincare. Bhelwa is known for its use in traditional remedies for skin diseases and neurological conditions, although caution is required due to its irritant properties.

Other moderately abundant species include *Ficus semicordata* Miq. (6.32%), *Bauhinia variegata* L. (5.95%), and *Ficus benghalensis* L. (5.95%). These species are used in treating digestive issues, diarrhea, and as spiritual plants in local customs. The frequent occurrence of these trees across the study area reflects both their ecological adaptability and integration into local healing systems.

*Azadirachta indica* A.Juss. (Neem) and *Aegle marmelos* (L.) Corrêa (Bel) recorded relative densities of 5.20% and 5.58%, respectively. Neem’s antimicrobial, anti-inflammatory, and wound-healing properties make it a cornerstone of traditional medicine. Similarly, Bel is considered sacred and is widely used for gastrointestinal ailments. Their moderate density may indicate sustainable use or ongoing regeneration due to cultivation and protection in sacred groves.

On the other hand, species such as *Cordia macleodii* (Griff.) Hook.f. & Thomson (0.74%), *Saraca asoca* (Roxb.) W.J.de Wilde (1.49%), and *Cordia dichotoma* G.Forst. (2.23%) showed relatively low-density values. This suggests either limited natural distribution or possible anthropogenic pressures. Despite their lower density, these species are highly valued for treating respiratory problems, gynecological issues, and inflammation, highlighting a gap between ecological availability and ethnomedicinal reliance^76^.

The data indicate that the species with high relative density are often those with multiple traditional uses, high demand, or wide ecological adaptability. Conversely, low-density species, although less visible in the landscape, may still hold vital ethnomedicinal significance. This underlines the need for targeted conservation strategies to ensure the continued availability of both abundant and rare species critical to tribal healthcare systems.

### Conservation significance of medicinal plant species

Assessment of the recorded plant species against the IUCN Red List (2025) revealed the presence of taxa of conservation concern. Notably, *Dalbergia latifolia* Roxb. was categorized as vulnerable, indicating potential risk from unsustainable harvesting due to its continued use in traditional practices. In contrast, several widely used species, including *Cordia macleodii* (Griff.) Hook.f. & Thomson, *Gmelina arborea* Roxb. ex Sm., *Madhuca longifolia* var. *latifolia* (Roxb.) A.Chev., *Melia azedarach* L., *Phyllanthus emblica* L., *Senegalia catechu* (L.f.) P.J.H.Hurter & Mabb., *Terminalia chebula* Retz., *Vitex negundo* L., and *Woodfordia fruticosa* (L.) Kurz, were listed as Least Concern (LC), suggesting relatively stable populations^77^.

Despite the use of a threatened species, no specific traditional conservation measures were documented among the informants. This absence highlights a critical need to raise awareness regarding the conservation status of ethnobotanically important species. The findings emphasize the importance of integrating conservation education and sustainable harvesting practices within local knowledge systems to safeguard biodiversity.

### Strategies for conservation and ethnobotanical knowledge development

To ensure the conservation and sustainable use of ethnobotanical knowledge among the Baiga tribe, it is essential to adopt both ecological and socio-cultural strategies. One promising approach is the establishment of Medicinal Plant Conservation and Development Areas (MPCDAs), which can serve as in situ conservation sites for regionally important medicinal species while also supporting community-led initiatives for traditional knowledge preservation^78^. Engaging tribal youth in ethnobotanical documentation programs, organizing training workshops on sustainable harvesting and cultivation practices, and integrating validated traditional knowledge into local primary healthcare systems are also recommended. Furthermore, including ethnobotanical concepts in local school curricula can foster early awareness and respect for traditional ecological knowledge among the younger generation. These integrated efforts can help strengthen both biodiversity conservation and community empowerment.

## Conclusion

The present study underscores the exceptional ethnobotanical knowledge of the Baiga tribe, showcasing their deep connection with nature and the sustainable use of medicinal plants. The documentation of 80 plant species, with high informant consensus factor values, highlights the tribe’s collective wisdom and its potential contributions to traditional and modern healthcare systems. The dominance of Fabaceae, the preference for bark as the primary plant part, and the reliance on paste preparation methods reflect unique cultural practices that warrant further exploration. Phytosociological analysis revealed that *Diospyros melanoxylon* Roxb. (IVI = 44.89) and *Shorea robusta* C.F.Gaertn. (IVI = 26.33) were the most ecologically dominant species, indicating their significant role in both forest structure and traditional medicine. The high IVI values of *Aegle marmelos* (L.) Corrêa, *Azadirachta indica* A.Juss., and *Terminalia bellirica* (Gaertn.) Roxb. further reinforce their medicinal and ecological importance. The strong correlation between ethnomedicinally valuable species and their prevalence in the forest highlights the need for conservation strategies that protect both biodiversity and indigenous knowledge systems. This research fills a critical gap in the ethnopharmacological literature by shedding light on the underrepresented medicinal traditions of the Baiga tribe, offering a comprehensive baseline for future studies. The findings not only emphasize the importance of preserving this invaluable cultural heritage but also point to significant opportunities for biodiversity conservation and the development of novel therapeutic agents.

To ensure long-term conservation and sustainable use of this knowledge, the establishment of Medicinal Plant Conservation and Development Areas (MPCDAs) is recommended. These areas can serve as in situ conservation zones for key medicinal species while promoting community-led preservation of traditional practices. Additional strategies such as involving tribal youth in documentation programs, organizing training workshops on sustainable harvesting, and integrating validated traditional remedies into local healthcare systems are also vital. Introducing ethnobotanical education at the school level may further encourage cultural pride and knowledge retention. These integrated efforts will not only protect biodiversity but also empower indigenous communities and support equitable benefit-sharing. As global attention increasingly shifts toward natural remedies and sustainable medicine, the Baiga tribe’s traditional practices stand as a testament to the richness of indigenous knowledge systems. This study calls for collaborative efforts among researchers, policymakers, and conservationists to safeguard these practices for the benefit of future generations.

## Data Availability

All data generated or analyzed during current study are available in the manuscript, figures, and tables.
